# Senescence‐Driven Remodeling Defines an Aggressive and Immunomodulatory Subtype of Endometriosis

**DOI:** 10.1111/acel.70463

**Published:** 2026-03-27

**Authors:** Jingchun Liu, Wuyue Han, Jianming Tang, Huanzhi Wan, Haoyu Wang, Jiaxin Peng, Wenjing Ma, Min Hu, Li Hong

**Affiliations:** ^1^ Department of Gynecology and Obstetrics Renmin Hospital of Wuhan University Wuhan Hubei China; ^2^ Department of Otolaryngology‐Head and Neck Surgery Renmin Hospital of Wuhan University Wuhan Hubei China

**Keywords:** endometriosis, macrophage, PAK4, senescence, stigmasterol

## Abstract

Endometriosis is a benign yet invasive disease characterized by ectopic endometrial growth and immune remodeling. While emerging evidence implicates cellular senescence in disease progression, the underlying mechanisms remain largely undefined. In this study, we identified marked heterogeneity in senescence across ectopic lesions and observed that P21‐activated kinase 4 (PAK4) was consistently upregulated in senescent cells. Mechanistically, senescence induced PAK4 expression, which in turn interacted with AKT and enhanced its phosphorylation, thereby activating the PI3K/AKT signaling pathway and further amplifying the senescence phenotype. This senescence‐PAK4‐AKT positive feedback loop ultimately promoted lesion aggressiveness and M2 macrophage polarization. Silencing PAK4 alleviated cellular senescence, attenuated lesion invasiveness, and suppressed immune remodeling. Notably, stigmasterol, a natural phytosterol, effectively downregulated PAK4 expression, disrupted the senescence‐AKT feedback loop, and consequently inhibited senescence, invasion, and M2 polarization both in vitro and in vivo. Together, our findings establish a senescence‐driven PAK4/AKT signaling circuit that fosters an aggressive, immunomodulatory endometriosis subtype and identify stigmasterol as a promising senescence‐targeted therapeutic agent.

## Graphical abstract

Senescence in ectopic endometrial cells promotes SASP‐mediated M2 macrophage polarization and enhanced invasiveness of endometriotic lesions. PAK4 drives this process via PI3K/AKT signaling, while stigmasterol suppresses PAK4 and alleviates senescence‐associated immune remodeling.

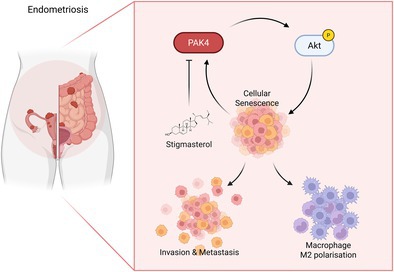

## 1 Introduction

Endometriosis is a prevalent gynecological disorder characterized by the presence of endometrial‐like tissue outside the uterine cavity, affecting approximately 190 million women worldwide. This condition significantly impairs reproductive health and overall quality of life, manifesting clinically as chronic pelvic pain, menstrual irregularities, and infertility (Zondervan et al. 2020; Liu et al. 2023). Current therapeutic strategies primarily involve hormonal suppression to inhibit ectopic endometrial proliferation, often supplemented by surgical excision when necessary. However, postoperative recurrence remains common, driven by factors such as persistent estrogen stimulation, incomplete lesion removal, immune dysfunction, genetic susceptibility, and the use of ovulation‐inducing agents. Although benign in histology, endometriosis exhibits malignant‐like behaviors, including invasion, metastasis, and recurrence, complicating its clinical management (Artemova et al. 2021).

Cellular senescence is a complex biological process characterized by stable cell cycle arrest accompanied by diverse phenotypes such as resistance to apoptosis, secretion of pro‐inflammatory factors, and promotion of tissue remodeling. While senescence traditionally serves as a tumor‐suppressive mechanism, accumulating evidence suggests that senescent cells can influence the surrounding microenvironment, fostering chronic inflammation and contributing to disease progression (Campisi and D'Adda 2007; Cui et al. 2024; Wang et al. 2024). Recent studies have identified the presence of senescent cells within endometriotic lesions, highlighting a potential but understudied dimension of disease pathology (Malvezzi, Dobo, Filippi, Mendes Do Nascimento, et al. 2022; Malvezzi, Dobo, Filippi, Mendes, et al. 2022). Despite its clinical importance, the characterization of senescence within endometriosis remains limited, as current classifications are largely based on lesion morphology and anatomical location. A deeper understanding of cellular senescence in endometriosis may thus facilitate more accurate disease stratification and inform the development of targeted therapeutic strategies.

P21‐activated kinase 4 (PAK4) is a conserved serine/threonine kinase that plays a critical role in regulating cell proliferation and adhesion. Recent studies have implicated PAK4 in tumor progression, epithelial‐mesenchymal transition (EMT), and immune modulation, primarily through the activation of signaling pathways such as ERK/MEK and PI3K/AKT, highlighting its importance in pathological tissue remodeling (Wang et al. 2025). However, the role of PAK4 in endometriosis remains largely unexplored.

In this study, through bioinformatics analysis, primary cell and animal experiments, we identified significant heterogeneity within ectopic endometrial tissues and distinguished senescent from non‐senescent subtypes based on a senescence‐associated signature. Senescent ectopic lesions exhibited enhanced invasiveness and increased M2 macrophage infiltration. Mechanistically, PAK4 was found to be upregulated in senescent lesions and associated with activation of the PI3K/Akt pathway. Furthermore, we demonstrated that stigmasterol effectively downregulated PAK4 expression, alleviated cellular senescence, reduced lesion aggressiveness, and attenuated M2 macrophage polarization both in vitro and in vivo. These findings highlight PAK4 as a potential therapeutic target in endometriosis and propose stigmasterol as a promising intervention.

## 2 Materials and Methods

### 2.1 Endometriosis Models and Animal Experiments

Female C57BL/6 mice (5 weeks old) were housed under specific pathogen‐free conditions at the Animal Breeding Center of Wuhan University People's Hospital and acclimatized for 1 week. Mice were randomly assigned to experimental groups and provided with irradiated standard chow and sterile water unless otherwise specified. An endometriosis donor–recipient transplantation model was established. Briefly, uterine horns from donor mice were excised, the myometrium was removed, and endometrial tissues (5 × 5 mm) were sutured onto the peritoneum of recipient mice. Sham‐operated mice served as controls. Postoperatively, mice received intramuscular estradiol (30 μg/kg) for three consecutive days to promote endometrial growth. Ectopic lesions were harvested at estrus 4 weeks after induction. For the senescence model, mice received intraperitoneal injections of D‐galactose (500 mg/kg) for 4 weeks after endometriosis induction, with PBS as control. For stigmasterol treatment, mice were administered stigmasterol (50 mg/kg/day) by oral gavage for 3 weeks, while control mice received corn oil. Experimental procedures and data analyses were performed in a double‐blind manner. All animal experiments were approved by the Laboratory Animal Ethics Committee of Renmin Hospital of Wuhan University and conducted in accordance with the Basel Declaration (No. WDRM‐20230410D).

### 2.2 Clinical Samples

Between September and December 2024, paired eutopic and ectopic endometrial tissues were collected from 10 patients (aged 30–40 years) with histologically confirmed endometriosis who underwent laparoscopic or open surgery. Fresh specimens were immediately frozen in liquid nitrogen and sectioned for subsequent histological and immunostaining analyses. Inclusion criteria were as follows: (1) age 30–40 years; (2) histologically confirmed endometriosis; (3) availability of paired eutopic and ectopic endometrial tissues of adequate quality; (4) no hormonal or immunomodulatory treatment within 3 months before surgery; (5) regular menstrual cycles (24–35 days) with documented last menstrual period; and (6) written informed consent. Exclusion criteria included: (1) coexisting gynecological diseases such as adenomyosis or ovarian tumors; (2) pregnancy, lactation, or abortion within the previous 3 months; (3) autoimmune or chronic infectious diseases; (4) prior radiotherapy, chemotherapy, or immunosuppressive therapy; (5) severe systemic or psychiatric disorders; or (6) inadequate tissue quality for downstream analyses. The study was approved by the Ethics Committee of Renmin Hospital of Wuhan University and conducted in accordance with the Declaration of Helsinki (WDRY2023‐K137).

### 2.3 Reagents, Antibodies, and Primers

All reagents were purchased from MedChemExpress, including estradiol (HY‐B0141), stigmasterol (HY‐N0131), D‐galactose (HY‐N0210), and corn oil (HY‐Y1888). Flag‐PAK4 and control plasmids were obtained from Miaoling Biotechnology (Wuhan, China).

Primary antibodies used in this study included NCAPH2 (ab200659, Abcam), PAK4 (14685‐1‐AP, Proteintech), HMGA1 (29895‐1‐AP, Proteintech), SOCS1 (ab280886, Abcam), OGT (11576‐2‐AP, Proteintech), BRD7 (51009‐2‐AP, Proteintech), AKT (10176‐2‐AP, Proteintech), phospho‐AKT (66444‐1‐Ig, Proteintech), ACTB (20536‐1‐AP, Proteintech), CD206 (60143‐1‐Ig, Proteintech), CD86 (13395‐1‐AP, Proteintech), and DYKDDDDK tag (20543‐1‐AP, Proteintech). Secondary antibodies included HRP‐conjugated goat anti‐mouse IgG (SA00001‐1, Proteintech), HRP‐conjugated goat anti‐rabbit IgG (SA00001‐2, Proteintech), CoraLite488‐conjugated goat anti‐mouse IgG (SA00013‐1, Proteintech), CoraLite488‐conjugated goat anti‐rabbit IgG (SA00013‐2, Proteintech), and CoraLite594‐conjugated goat anti‐rabbit IgG (SA00013‐4, Proteintech).

The sequences of the PAK4‐targeting siRNAs were as follows: si‐PAK4#1, 5′‐GAGCCUGAUUGAGGAAUCA‐3′; si‐PAK4#2, 5′‐GUGUUUGGGAAGAGGAAGAA‐3′; si‐NCAPH2, 5′‐AAGUUGCAGGACUUCCACAAG‐3′; si‐SOCS1, 5′‐CAGCCAGUUUAG GUAAUAA‐3′; si‐HMGA1,5′‐CUCCUGAAUUUGCCUGUA U‐3′. The primers used in the study were sourced from Sangon Biotech, and the specific sequences can be found in Table S1.

### 2.4 Cell Extraction, Culture and Treatment

Ectopic endometrial tissues were dissected under a stereomicroscope and digested with 0.1% type II collagenase (C2‐BIOC, Sigma) at 37°C for 30 min. The cell suspension was centrifuged, and the pellet was resuspended in complete medium consisting of DMEM (Gibco), 20% fetal bovine serum (FBS; Gibco), and 1% penicillin–streptomycin (Gibco) and cultured at 37°C in 5% CO_2_. After formation of confluent monolayers, mouse endometrial epithelial cells (MEECs) and mouse endometrial stromal cells (MESCs) were separated and purified by differential mechanical dissociation. Cells were subsequently maintained in complete medium. For senescence induction, cells were treated with D‐galactose (20 mg/mL) for 48 h as previously described (Wan et al. 2023). RAW264.7 macrophages were kindly provided by the Research Institute of Otolaryngology–Head and Neck Surgery, Renmin Hospital of Wuhan University. RAW264.7 cells were cultured in high‐glucose DMEM supplemented with 10% FBS at 37°C in 5% CO_2_. For stigmasterol treatment, cells were exposed to stigmasterol (8 μg/mL) for 24 h unless otherwise indicated.

### 2.5 Conditioned Medium Preparation

Conditioned medium (CM) was prepared from MEECs and MESCs under non‐senescent or senescent conditions. For senescence induction, cells were treated with D‐galactose (20 mg/mL) for 48 h. After treatment, cells were washed twice with PBS and cultured in fresh medium for 24 h to generate CM, thereby minimizing carryover of D‐galactose or other treatment reagents. For PAK4 knockdown–derived CM, cells were transfected with siRNAs targeting PAK4 or control siRNA prior to senescence induction. After D‐galactose treatment, cells were washed twice with PBS and incubated in fresh medium for CM collection to avoid residual siRNA transfection reagents. For stigmasterol‐derived CM, senescent cells were treated with stigmasterol (8 μg/mL) for 24 h, followed by two PBS washes and incubation in fresh medium for CM collection to minimize drug carryover. Supernatants were collected after 24 h, centrifuged at 1000×*g* for 10 min at 4°C to remove cell debris, and passed through a 0.22‐μm filter. CM was used immediately or stored at −80°C until use. For IL‐6 neutralization experiments, CM was pre‐incubated with an anti‐IL‐6 neutralizing antibody for 1 h at 37°C before macrophage stimulation. An isotype‐matched IgG antibody was used as the control.

### 2.6 Western Blot

Proteins were extracted from tissues and cells using pre‐cooled RIPA buffer (G2002, Servicebio) and separated by SDS–PAGE, followed by transfer onto PVDF membranes. After blocking with 5% skim milk, membranes were incubated with primary antibodies overnight at 4°C. After incubation with secondary antibodies, protein bands were visualized using enhanced chemiluminescence reagent (Epizyme, SQ101L) and imaged with a ChemiDoc system (Bio‐Rad).

### 2.7 Cell Migration and Invasion Assays

Cell migration and invasion of MEECs and MESCs were assessed using Transwell assays. For migration assays, cells (4 × 10^4^ per well) were seeded in the upper chambers with DMEM containing 1% FBS, while the lower chambers were filled with CM as indicated. After incubation for 48 h at 37°C, cells were fixed with 4% paraformaldehyde for 15 min and stained with 0.1% crystal violet for 15 min. Non‐migrated cells on the upper surface were removed, and migrated cells were imaged and counted under a microscope. For invasion assays, the upper chambers were pre‐coated with Matrigel (354237, Corning), and the remaining procedures were identical to those used for migration assays.

### 2.8 Co‐Immunoprecipitation

As previously described, immunoprecipitation was performed using Protein A/G agarose beads (Biolinkedin) (Liu et al. 2024). A total of 1 × 10^7^ cells were collected and lysed in IP lysis buffer. The antibody‐preconjugated magnetic beads were added to the cell lysate and incubated overnight at 4°C. On the following day, the immune complexes were collected and washed three times with IP lysis buffer. The final bead complexes were boiled in SDS loading buffer and subjected to western blot analysis for protein detection.

### 2.9 SA‐β‐Gal Staining

For primary cells, cells were inoculated in six‐well plates and cultured until the cells were in good condition but did not show contact inhibition. Cells were then stained using the SA‐β‐gal kit (G1580, Solarbio) according to the manufacturer's instructions and incubated overnight at 37°C in a CO2‐free environment. Staining was observed under a microscope the following day and images were taken. For tissue cryosections, the staining procedures were performed as previously described (Wan et al. 2024).

### 2.10 Real‐Time Quantitative PCR (qPCR)

Total RNA was extracted from tissues or cells using TRIzol reagent (Takara) according to the manufacturer's instructions. RNA was isolated by chloroform extraction and isopropanol precipitation, followed by washing with absolute ethanol. cDNA was synthesized using Hifair III 1st Strand cDNA Synthesis SuperMix (Yeasen). Quantitative PCR was performed using SYBR Green Master Mix (No Rox, Yeasen) on a CFX Connect Real‐Time PCR system (Bio‐Rad). Relative mRNA expression levels were calculated using the Livak (2^−ΔΔCt^) method. Primer sequences are listed in Table S1.

### 2.11 Elisa

The concentrations of IL‐1α (Elabscience), IL‐1β (Elabscience), IL‐6 (Elabscience), and IL‐8 (Absin) were measured using commercial ELISA kits according to the manufacturers' instructions. Absorbance at 450 nm was measured using a microplate reader, and cytokine concentrations were calculated based on standard curves.

### 2.12 Flow Cytometry

Mouse spleens were mechanically dissociated to obtain single‐cell suspensions. After centrifugation, erythrocytes were lysed using red blood cell lysis buffer (Elabscience), and cells were washed and resuspended for staining. Fc receptors were blocked using FcR blocking reagent (Elabscience), followed by incubation with FITC‐conjugated anti‐CD11b, PerCP/Cy5.5‐conjugated anti‐F4/80, PE‐conjugated anti‐CD86, and APC‐conjugated anti‐CD206 antibodies to identify macrophage subsets. RAW264.7 cells were processed using the same staining procedure beginning with FcR blocking. Flow cytometry was performed using a CytoFLEX flow cytometer (Beckman Coulter), and data were analyzed with CytExpert software.

### 2.13 Immunofluorescence

Immunofluorescence staining of frozen tissue sections was performed as previously described (Wan et al. 2024). Macrophages were stained with anti‐CD86 and anti‐CD206 antibodies to identify M1 and M2 phenotypes, respectively, followed by incubation with CoraLite594‐conjugated goat anti‐rabbit IgG (H + L) and CoraLite488‐conjugated goat anti‐mouse IgG (H + L). Nuclei were counterstained with DAPI (Servicebio). MEECs and MESCs were fixed and incubated with primary antibodies against PAK4 and AKT, followed by species‐specific fluorescent secondary antibodies. Fluorescence images were acquired using a fluorescence microscope.

### 2.14 Datasets and Differential Gene Analysis

Gene expression datasets comparing eutopic and ectopic endometrium (GSE7305, GSE11691, GSE25628, GSE51981, and GSE120103) were obtained from the Gene Expression Omnibus (GEO) database. Senescence‐associated genes were retrieved from the CellAge database (accessed August 2023), and 217 experimentally validated non‐tumor senescence‐related genes were included for subsequent analyses (Table S2). Herbal compound data were obtained from the HERB database (accessed August 2023). Differential gene expression analysis was performed using GEO2R with an adjusted *p* value < 0.05 and |log_2_FC| > 1. Volcano plots were generated to visualize differentially expressed genes, and heatmaps were constructed using the top 50 genes ranked by adjusted *p* value. UpSet plots were used to identify overlapping genes across datasets. Because the datasets were derived from different platforms, cross‐platform batch correction was not performed.

### 2.15 Consensus Clustering

Consensus clustering was performed on the normalized expression matrix of 217 senescence‐related genes in the GSE51981 dataset using the ConsensusClusterPlus package to identify molecular subtypes. The maximum number of clusters (*K*) was set to 10, and the optimal cluster number was determined based on the cumulative distribution function (CDF) curves and cluster consensus scores. *K* = 2 was selected as the optimal clustering solution due to the highest within‐cluster consistency.

### 2.16 Enrichment Analysis and Functional Annotation

Gene enrichment analysis and functional annotation were performed using the clusterProfiler package. Kyoto Encyclopedia of Genes and Genomes (KEGG) pathways and Gene Ontology (GO) terms were analyzed, and the top five enriched terms ranked by adjusted *p* value were visualized for each category. For gene set enrichment analysis (GSEA), gene sets were obtained from the msigdbr package and enrichment analysis was conducted using clusterProfiler.

### 2.17 Signature Construction and Analysis

A senescence signature was constructed using least absolute shrinkage and selection operator (LASSO) regression implemented in the glmnet package with 10‐fold cross‐validation. Six genes and their corresponding coefficients at the optimal lambda (minimum mean cross‐validation error) were selected to establish the senescence score model. The senescence score was calculated as follows: Senescence score = 1.77 × exp.(PAK4) + 0.62 × exp.(NCAPH2) + 0.37 × exp.(HMGA1) + 0.77 × exp.(SOCS1) + (−1.45) × exp.(OGT) + (−1.24) × exp.(BRD7).

Samples were stratified into high‐ and low‐senescence groups according to the median senescence score.

### 2.18 Immune Infiltration Analysis

Immune infiltration was evaluated using the ESTIMATE package, and stromal, immune, and ESTIMATE scores were calculated. The relative proportions of 22 immune cell types were estimated using the CIBERSORT algorithm (accessed August 2023).

### 2.19 Molecular Docking

The three‐dimensional structure of the target protein was obtained from the AlphaFold database (accessed October 2023). The molecular structure of stigmasterol (CID: 5280794) was downloaded from PubChem. Molecular docking was performed using AutoDock, and the optimal binding conformation was selected based on binding energy. Docking results were visualized using PyMOL.

### 2.20 Cell Viability Assay

Cell viability was assessed using a Cell Counting Kit‐8 (40203ES60, Yeasen) according to the manufacturer's instructions. After incubation with CCK‐8 reagent for 30 min at 37°C, absorbance at 450 nm was measured using a microplate reader, and relative cell viability was calculated.

### 2.21 Statistical Analysis

Data visualization and statistical analyses were performed using GraphPad Prism 9 and R software (version 4.1.0). Receiver operating characteristic (ROC) analyses were conducted using the pROC package. Calibration curves were generated using the rms package, and model calibration was evaluated using the ResourceSelection package. Pearson correlation analysis was used to assess associations between variables. All experiments were performed with at least three independent biological replicates. Comparisons between two groups were conducted using Student's *t*‐test, and multiple‐group comparisons were performed using one‐way ANOVA. The *p* value < 0.05 was considered statistically significant (*p* < 0.05, *p* < 0.01, *p* < 0.001; ns, not significant).

## 3 Result

### 3.1 Ectopic Endometrium Exhibits Reduced Enrichment of Senescence‐Associated Signatures

**FIGURE 1:**
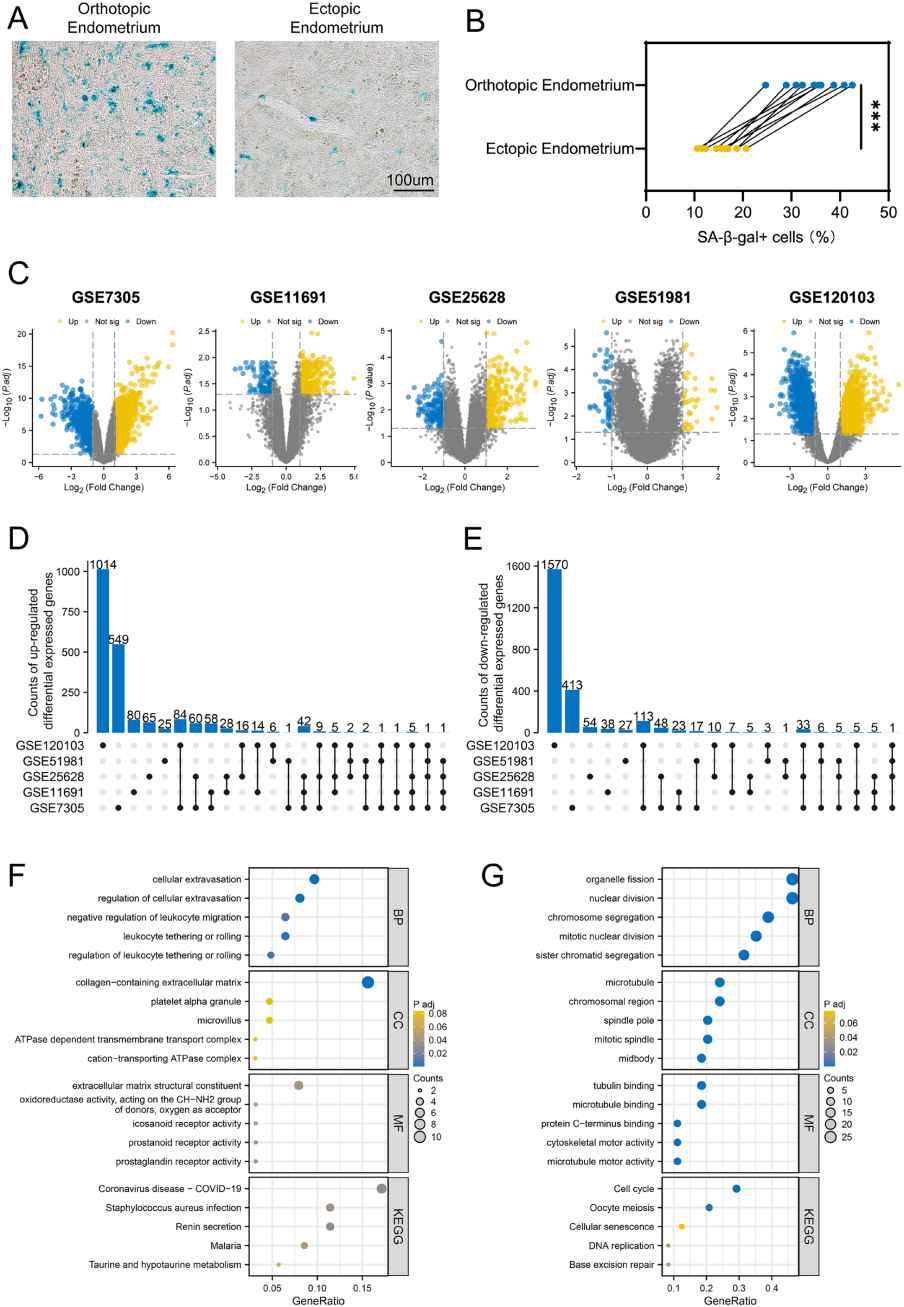
Genetic differences in orthotopic and ectopic endometrium. (A) SA‐β‐gal staining of orthotopic and ectopic endometrial samples from patients. (B) Proportion of SA‐β‐gal positive cells in clinical samples. (C) Volcano maps of differential genes in orthotopic and ectopic endometrium in GSE7305, GSE11691, GSE25628, GSE51981, and GSE120103. (D, E) Overlap of (D) upregulated and (E) downregulated differentially expressed genes in ectopic endometrial samples across multiple datasets. (F, G) Enrichment analysis of differentially expressed genes that were (F) upregulated and (G) downregulated in at least three datasets. ***, *p* < 0.001.

Previous studies have suggested the presence of senescent subpopulations in menstrual effluent‐derived endometrial tissues from patients with endometriosis, which are associated with cellular adhesion and dissemination (Shih et al. 2022). To evaluate senescence status in vivo, we collected paired eutopic (*n* = 10) and ectopic (*n* = 10) endometrial tissues from patients with endometriosis. SA‐β‐gal staining revealed that ectopic endometrium exhibited lower senescence levels compared with eutopic tissues (Figure 1A,B).

To further characterize molecular differences between eutopic and ectopic endometrium, we analyzed transcriptomic profiles from five independent GEO datasets (GSE7305, GSE11691, GSE25628, GSE51981, and GSE120103). Volcano plots illustrate differentially expressed genes across the datasets (Figure 1C). Genes showing consistent expression changes in at least three datasets were retained for integrative analysis. Using eutopic endometrium as the reference, 91 upregulated and 62 downregulated genes were identified in ectopic tissues (Figure 1D,E).

Functional enrichment analysis showed that upregulated genes were mainly associated with cellular extravasation, collagen‐containing extracellular matrix, and extracellular matrix structural components (Figure 1F). In contrast, downregulated genes were significantly enriched in pathways related to the cell cycle, DNA replication, and cellular senescence (Figure 1G).

These findings indicate that ectopic endometrium exhibits reduced enrichment of senescence‐associated signatures compared with eutopic tissues.

### 3.2 Ectopic Endometrial Lesions Exhibit Heterogeneous Senescence States

To determine whether senescence heterogeneity exists in ectopic endometrium, we retrieved 217 non‐tumor senescence‐associated genes from the CellAge database and performed unsupervised clustering analysis using the GSE51981 dataset. Ectopic endometrial samples were stratified into two major clusters (C1 and C2) (Figure S1A), which were clearly separated by principal component analysis (Figure S1B). Differential expression analysis revealed distinct transcriptional profiles between the two clusters (Figure S1C,D).

GSEA was performed to characterize functional differences between clusters. Genes downregulated in C2 were enriched in pathways related to focal adhesion, ECM‐receptor interaction, regulation of actin cytoskeleton, and cell cycle (Figure S1E). In contrast, genes upregulated in C2 were enriched in oxidative phosphorylation, apoptosis, purine metabolism, antigen processing and presentation, and endometrial cancer pathways (Figure S1F). These results suggest that C2 exhibits features consistent with cell cycle arrest, a hallmark of cellular senescence. Enrichment of endometrial cancer‐related pathways further indicates a more aggressive phenotype in the high‐senescence subgroup.

**FIGURE 2:**
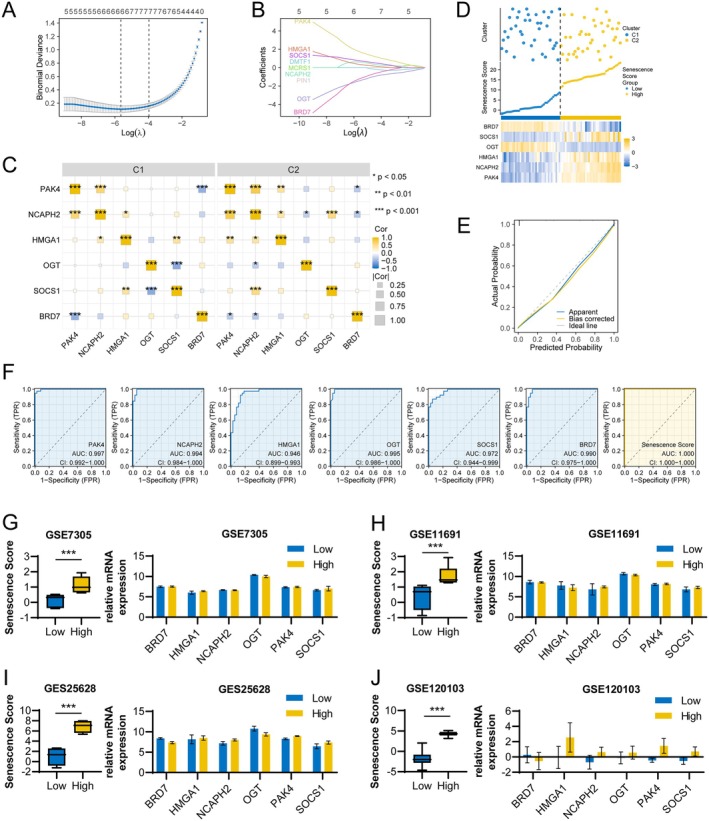
Senescence scores distinguish ectopic endometrial subgroups. (A) LASSO regression analysis of 217 senescence‐related genes. (B) LASSO variable trace plots. (C) Correlation of mRNA levels of six genes in clusters 1 and 2. (D) Subgroup differentiation, senescence scores and gene expression profiles based on the median senescence score of the GSE51981 cohort. (E) Diagnostic calibration curves for senescence scores. Closer to the diagonal line indicates a better fit. (F) ROC curves for PAK4, NCAPH2, HMGA1, OGT, SOCS1, BRD7 and senescence scores in the GSE51981 cohort. (G–J) Grouping of ectopic endometrium based on senescence score and PAK4, NCAPH2, HMGA1, OGT, SOCS1, BRD7 gene expression levels in the (G) GSE7305 dataset, (H) GSE11691 dataset, (I) GSE25628 dataset, and (J) GSE120103 dataset. *, *p* < 0.05; **, *p* < 0.01; ***, *p* < 0.001.

To quantify senescence heterogeneity, we constructed a senescence signature using LASSO regression based on the 217 senescence‐related genes (Figure 2A,B). The senescence score was calculated as follows: Senescence score = 1.77 × exp.(PAK4) + 0.62 × exp.(NCAPH2) + 0.37 × exp.(HMGA1) + 0.77 × exp.(SOCS1) + (−1.45) × exp.(OGT) + (−1.24) × exp.(BRD7). Among the six signature genes, PAK4 showed the largest coefficient, indicating its dominant contribution to the senescence score. Expression of SOCS1, HMGA1, NCAPH2, and PAK4 positively correlated with the senescence score, whereas BRD7 and OGT showed negative correlations (Figure 2C,D). The senescence score clearly distinguished C1 and C2 clusters, and calibration analysis demonstrated good predictive performance (Figure 2E).

Receiver operating characteristic (ROC) analysis showed that the senescence score outperformed individual genes in discriminating senescence subtypes (Figure 2F). The robustness of the senescence signature was further validated in four independent datasets, where high‐score samples consistently exhibited similar expression patterns compared with low‐score samples (Figure 2G–J).

### 3.3 Senescence‐Enriched Endometriotic Lesions Exhibit Enhanced Invasive and Migratory Capabilities

To evaluate the functional relevance of the senescence score, a mouse model of endometriosis was established. Senescence was induced in ectopic lesions by intraperitoneal injection of D‐galactose, generating a senescence‐enriched microenvironment. Senescent ectopic lesions exhibited significantly larger volumes than non‐senescent controls (Figure S2).

**FIGURE 3:**
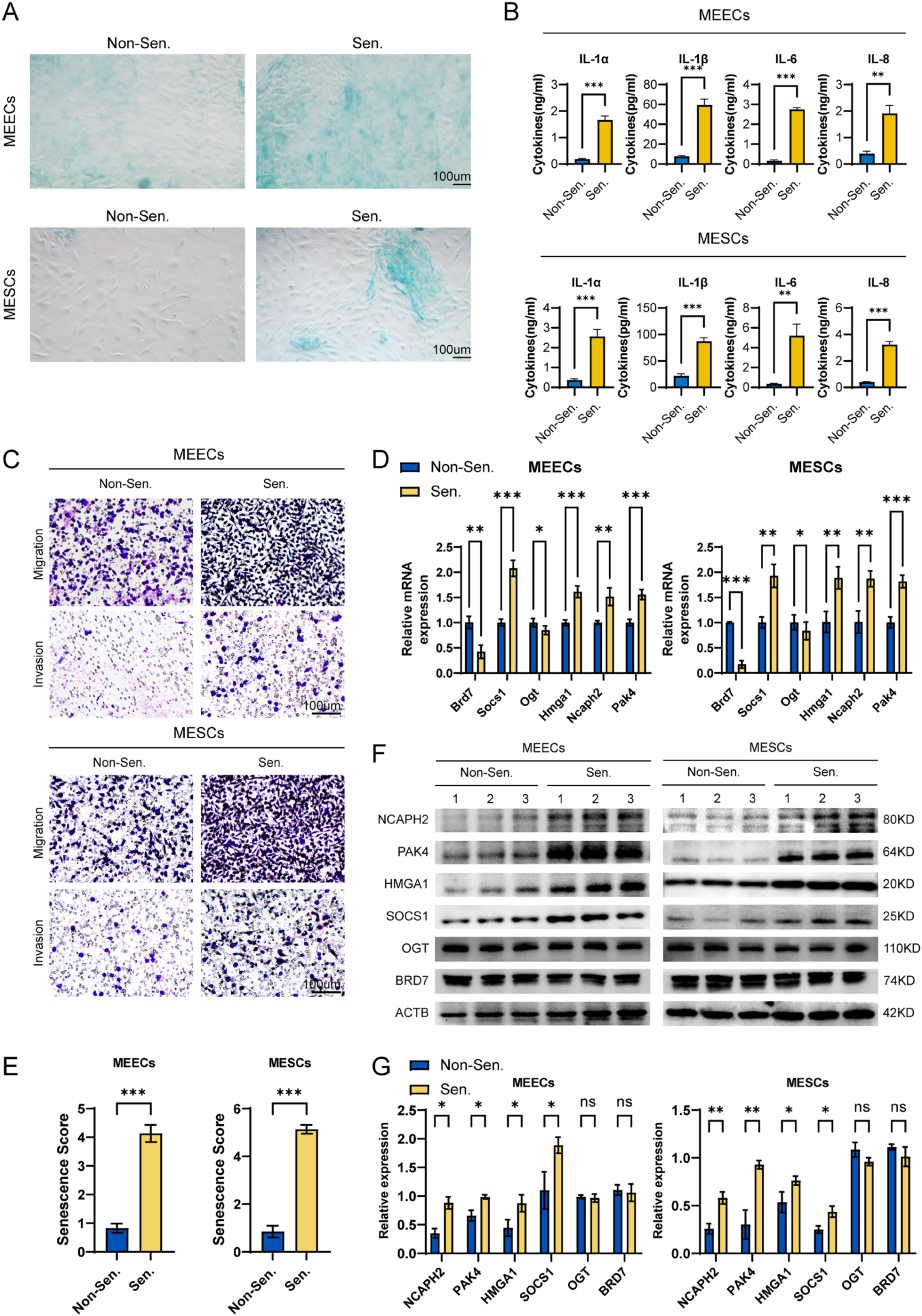
Senescent MEECs and MESCs demonstrated significantly increased migration and invasion, accompanied by higher senescence scores. (A) Senescence was induced in MEECs and MESCs using β‐gal treatment, followed by SA‐β‐gal staining. (B) Quantification of SASP factor secretion in MEECs and MESCs. (C) Assessment of migratory and invasive capacities of MEECs and MESCs using Transwell assays. (D) mRNA expression levels of Brd7, Socs1, Ogt, Hmga1, Ncaph2, and Pak4 in non‐senescent and senescent MEECs and MESCs. (E) Senescence scores of non‐senescent and senescent MEECs and MESCs. (F) Representative Western blot images showing BRD7, SOCS1, OGT, HMGA1, NCAPH2, and PAK4 protein levels in non‐senescent and senescent MEECs and MESCs. (G) Quantification of protein expression levels of BRD7, SOCS1, OGT, HMGA1, NCAPH2, and PAK4 in non‐senescent and senescent cells. *, *p* < 0.05; **, *p* < 0.01; ***, *p* < 0.001; ns, not significant.

Primary ectopic endometrial cells were isolated and separated into mouse endometrial epithelial cells (MEECs) and stromal cells (MESCs). Senescent cells showed stronger SA‐β‐gal staining, confirming successful senescence induction (Figure 3A). Consistently, concentrations of key senescence‐associated secretory phenotype (SASP) factors, including IL‐1α, IL‐1β, IL‐6, and IL‐8, were significantly increased in the culture supernatants of senescent cells (Figure 3B).

Functionally, both senescent MEECs and MESCs displayed significantly enhanced migratory and invasive capacities compared with controls (Figure 3C). To determine whether the senescence signature reflects cellular senescence status, expression of signature genes was examined by qPCR and corresponding senescence scores were calculated. Senescent cells exhibited increased expression of Socs1, Hmga1, Ncaph2, and Pak4 and reduced expression of Brd7 and Ogt (Figure 3D), resulting in significantly higher senescence scores (Figure 3E).

Protein‐level analyses further confirmed elevated expression of SOCS1, HMGA1, NCAPH2, and PAK4 in both MEECs and MESCs (Figure 3F,G). In contrast, BRD7 and OGT protein levels showed no significant reduction.

### 3.4 Senescent Lesions Are Associated With Increased M2 Macrophage Infiltration

Given the association between senescence and lesion aggressiveness, we next investigated whether senescence influences the immune microenvironment in endometriotic lesions. ESTIMATE analysis of the GSE51981 dataset showed comparable stromal scores between high‐ and low‐senescence groups (Figure S3A), whereas immune scores and overall ESTIMATE scores were significantly higher in the high‐senescence group (Figure S3B,C), indicating enhanced immune infiltration. To further characterize the immune landscape, immune cell composition was estimated using the CIBERSORT algorithm (Figure S3D). Notably, the proportion of M2 macrophages was significantly increased in samples with high senescence scores (Figure S3E,F). Similar trends were observed in the GSE120103 and GSE11691 datasets (Figure S4A–D), whereas no significant differences were detected in GSE7305 or GSE25628 (Figure S4E–H).

**FIGURE 4:**
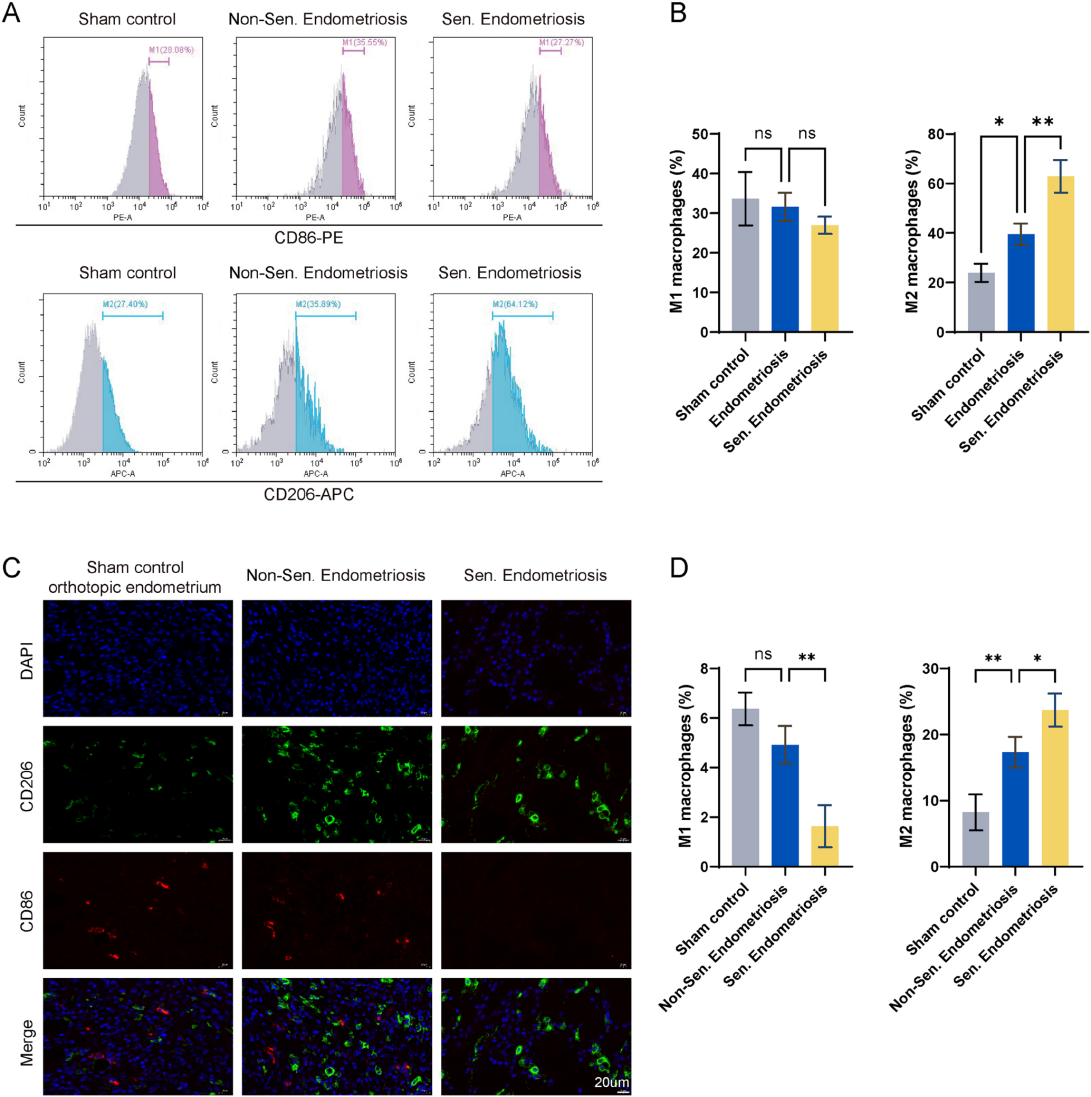
Senescent ectopic endometrium is associated with elevated levels of M2 macrophages. (A, B) (A) Flow cytometric analysis of macrophages and (B) quantification of M1 and M2 macrophage proportions in the spleens of mice from the sham‐operated, non‐senescent ectopic endometrium, and senescent endometrium groups. (C, D) (C) Immunofluorescence staining and (D) quantification of M1 and M2 macrophage proportions in orthotopic endometrium, non‐senescent ectopic endometrium, and senescent ectopic endometrium of the sham‐operated group. *, *p* < 0.05; **, *p* < 0.01; ns, not significant.

To validate these findings in vivo, we examined macrophage polarization in the endometriosis mouse model. Mice bearing ectopic lesions exhibited a higher proportion of splenic M2 macrophages than sham‐operated controls. Importantly, mice with senescent ectopic lesions showed further increases in M2 macrophage polarization compared with non‐senescent lesions (Figure 4A,B). We next assessed macrophage infiltration within ectopic lesions. Ectopic endometrium contained a higher proportion of M2 macrophages than eutopic endometrium (Figure 4C,D). Moreover, senescent ectopic lesions exhibited the highest levels of M2 macrophage infiltration. These findings indicate that senescence‐enriched ectopic endometrium is associated with increased M2 macrophage polarization.

### 3.5 PAK4 Regulates Senescence‐Associated Aggressive and Immunomodulatory Phenotypes in Ectopic Endometrial Cells

To determine whether senescent ectopic endometrial cells directly promote macrophage polarization, CM collected from non‐senescent and senescent MEECs and MESCs after removal of residual treatments were applied to RAW264.7 macrophages. CM from senescent cells markedly increased the proportion of CD206^+^ macrophages compared with non‐senescent controls. Neutralization of IL‐6 partially attenuated this effect, indicating that senescence‐associated secretory factors contribute to M2 polarization (Figure S5A,B).

To identify upstream regulators of the senescence‐associated program, we prioritized candidate genes based on the senescence signature model. Among the six signature genes, PAK4 exhibited the largest positive coefficient, suggesting a dominant contribution to senescence‐associated phenotypes.

**FIGURE 5:**
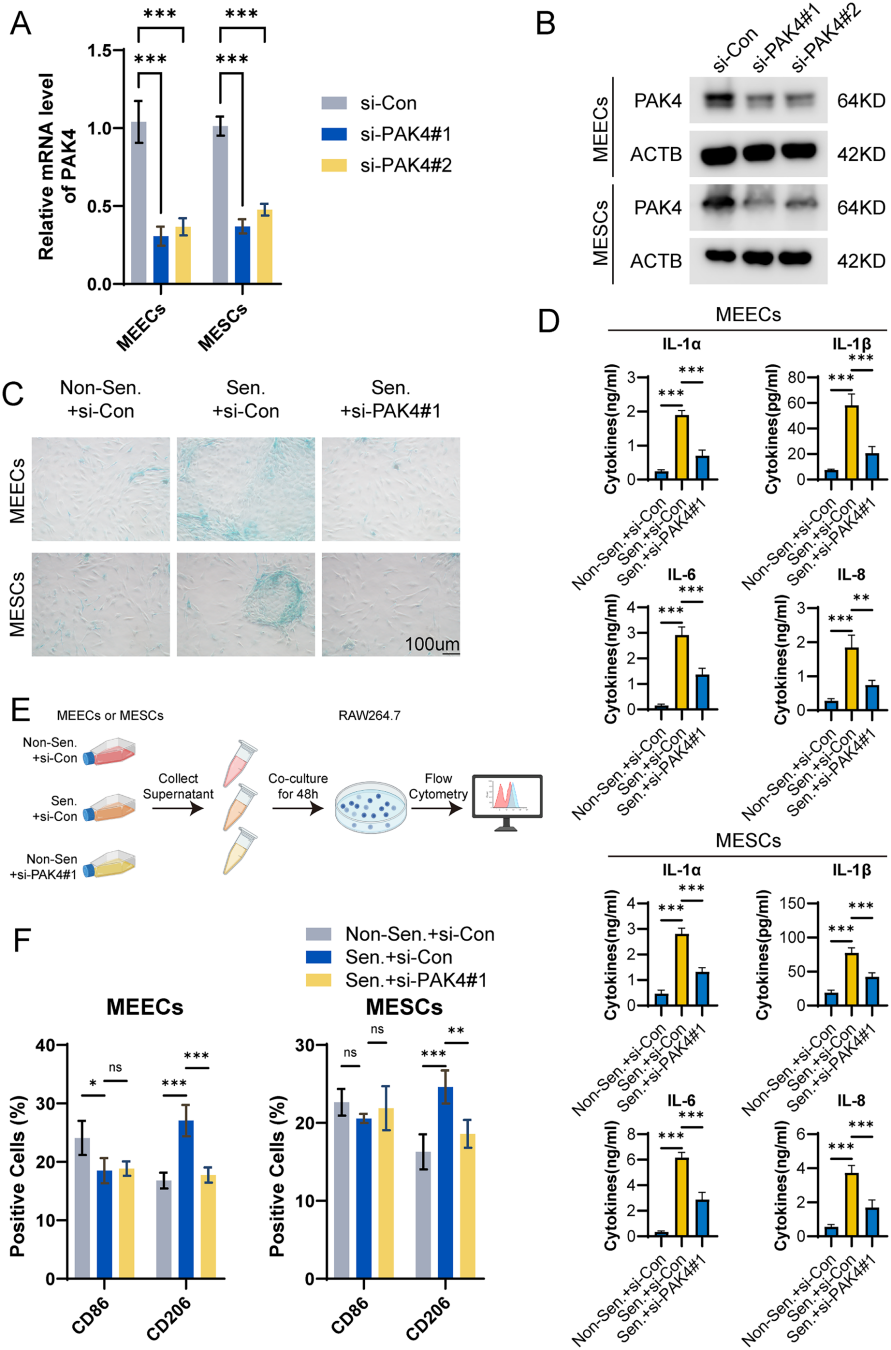
PAK4 regulates ectopic endometrial cell senescence and the associated macrophage M2 polarization. (A, B) PAK4 knockdown efficiency in MEECs and MESCs confirmed by qRT‐PCR (A) and Western blot (B). (C) SA‐β‐gal staining showing reduced senescence upon PAK4 silencing under D‐gal treatment. (D) ELISA analysis of SASP factors (IL‐1α, IL‐1β, IL‐6, IL‐8) in senescent cells with or without PAK4 knockdown. (E) Schematic of co‐culture: RAW264.7 macrophages were treated with CM from MEECs/MESCs after senescence induction and/or PAK4 knockdown. (F) Flow cytometry showing increased CD206^+^ M2 macrophages in response to senescent cell media, which was attenuated by PAK4 knockdown. *, *p* < 0.05; **, *p* < 0.01; ***, *p* < 0.001; ns, not significant.

We next examined the functional impact of PAK4 depletion in MEECs and MESCs. siRNA‐mediated knockdown efficiently reduced PAK4 mRNA and protein levels (Figure 5A,B). PAK4 depletion significantly attenuated D‐galactose‐induced cellular senescence, as evidenced by reduced SA‐β‐gal staining (Figure 5C). Consistently, secretion of senescence‐associated secretory phenotype (SASP) factors, including IL‐1α, IL‐1β, IL‐6, and IL‐8, was markedly decreased following PAK4 knockdown (Figure 5D). Moreover, PAK4 depletion significantly suppressed the migratory and invasive capacity of senescent MEECs and MESCs (Figure S6).

To determine whether other signature genes exert similar effects, SOCS1, HMGA1, and NCAPH2 were individually silenced in senescent MEECs and MESCs. Knockdown of these genes produced limited effects on senescence phenotypes (Figure S7), supporting a dominant role for PAK4 in regulating senescence‐associated programs.

We next examined whether PAK4‐dependent senescence influences macrophage polarization. CM from senescent MEECs and MESCs markedly increased the proportion of CD206^+^ RAW264.7 macrophages, whereas CM from PAK4‐depleted senescent cells significantly attenuated M2 polarization (Figure 5E,F). These results identify PAK4 as a key regulator of senescence‐associated aggressive and immunomodulatory phenotypes in ectopic endometrial cells.

### 3.6 PAK4 Promotes Cellular Senescence via Activation of PI3K/Akt Pathway

To elucidate the molecular mechanism by which PAK4 regulates senescence in ectopic endometrial cells, we re‐analyzed the GSE51981 dataset. GSEA was performed in senescent versus non‐senescent samples and in PAK4‐high versus PAK4‐low samples. Overlapping enriched pathways highlighted activation of PI3K/AKT signaling, suggesting a potential role of this pathway in PAK4‐associated senescence (Figure S8). Previous studies have shown that the PI3K/AKT signaling axis integrates key processes involved in cellular senescence (Bhatt et al. 2025), and PAK4 has been reported to regulate PI3K/AKT signaling (Chi et al. 2024). These findings prompted us to investigate whether PAK4 promotes senescence through PI3K/AKT activation in ectopic endometrial cells.

**FIGURE 6:**
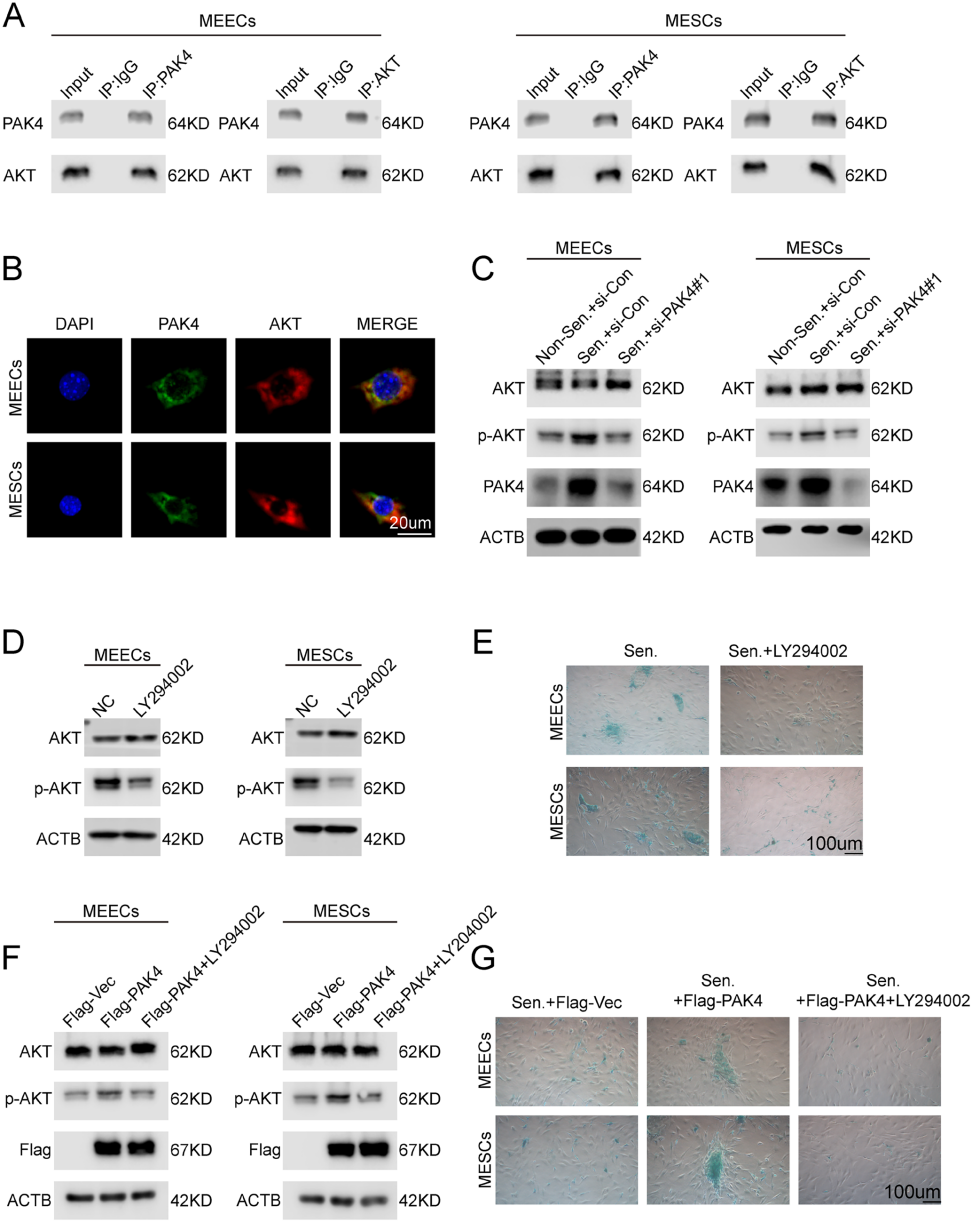
PAK4 promotes cellular senescence through activation of the PI3K/AKT signaling pathway. (A) Co‐IP analysis showing interaction between endogenous PAK4 and AKT in MEECs and MESCs. (B) Immunofluorescence images demonstrating colocalization of PAK4 and AKT in MEECs and MESCs. (C) Western blot showing decreased AKT phosphorylation following PAK4 knockdown under D‐gal‐induced senescence conditions. (D, E) Western blot showing that LY294002 treatment inhibits AKT phosphorylation. (E) SA‐β‐gal staining indicating that LY294002 reduces cellular senescence in MEECs and MESCs. (F) Western blot showing that LY294002 blocks AKT phosphorylation even in the presence of PAK4 overexpression. (G) SA‐β‐gal staining showing that LY294002 reverses PAK4 overexpression‐induced senescence. *, *p* < 0.05; **, *p* < 0.01; ***, *p* < 0.001; ns, not significant.

Co‐immunoprecipitation assays demonstrated an endogenous interaction between PAK4 and AKT (Figure 6A). Immunofluorescence staining further confirmed colocalization of PAK4 and AKT in both MEECs and MESCs (Figure 6B). Consistently, Western blot analysis showed that D‐galactose‐induced senescence was accompanied by increased AKT phosphorylation, whereas PAK4 knockdown markedly reduced AKT phosphorylation in both cell types (Figure 6C).

To determine the functional involvement of PI3K/AKT signaling in PAK4‐mediated senescence, MEECs and MESCs were treated with the PI3K inhibitor LY294002. LY294002 treatment significantly reduced the proportion of SA‐β‐gal–positive cells, phenocopying the effect of PAK4 knockdown (Figure 6D,E). Moreover, LY294002 treatment abolished the pro‐senescent effect induced by PAK4 overexpression (Figure 6F,G).

These findings support a model in which PAK4 promotes cellular senescence through activation of PI3K/AKT signaling.

### 3.7 Stigmasterol Suppresses PAK4 Expression and Alleviates Senescence and M2 Infiltration

**FIGURE 7:**
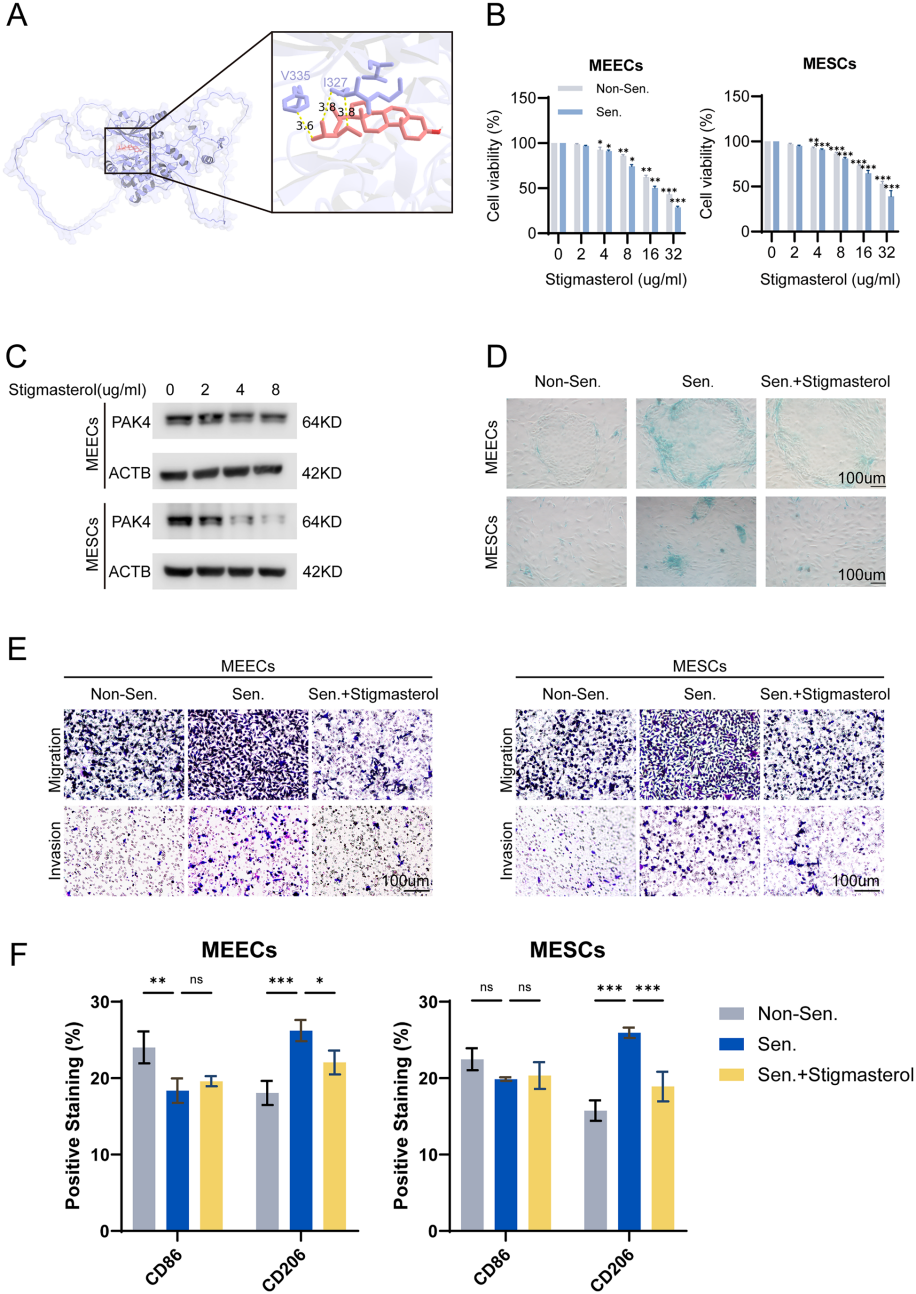
Stigmasterol alleviates cellular senescence and immune remodeling by targeting PAK4. (A) Molecular docking predicts strong binding between stigmasterol and PAK4. (B) Stigmasterol reduces the viability of senescent MEECs and MESCs in a dose‐dependent manner. (C) Western blot shows dose‐dependent downregulation of PAK4 protein levels. (D) SA‐β‐gal staining shows reduced senescence upon stigmasterol treatment. (E) Transwell assay indicates decreased migration and invasion. (F) CM from stigmasterol‐treated cells reduces M2 polarization of RAW264.7 macrophages. *, *p* < 0.05; **, *p* < 0.01; ***, *p* < 0.001; ns, not significant.

Given the central role of PAK4 in regulating senescence‐associated phenotypes, we next sought to identify small molecules with potential to target PAK4. Molecular docking analysis predicted that stigmasterol exhibits favorable binding affinity to the catalytic domain of PAK4 (Figure 7A). To validate this prediction, stigmasterol was applied to D‐galactose‐induced senescent MEECs and MESCs. Stigmasterol reduced the viability of senescent MEECs and MESCs in a dose‐dependent manner (Figure 7B). Western blot analysis further showed that stigmasterol downregulated PAK4 protein expression in a dose‐dependent fashion (Figure 7C). Treatment with 8 μg/mL stigmasterol significantly reduced both cell viability and PAK4 expression and was therefore used for subsequent experiments.

Functionally, stigmasterol significantly decreased the proportion of SA‐β‐gal–positive cells and suppressed the migratory and invasive capacities of MEECs and MESCs (Figure 7D,E), indicating attenuation of senescence‐associated aggressive phenotypes. To determine whether stigmasterol affects macrophage polarization, CM from stigmasterol‐treated senescent MEECs and MESCs was applied to RAW264.7 macrophages. Flow cytometry analysis showed that stigmasterol‐treated CM significantly reduced the proportion of CD206^+^ macrophages compared with senescent controls (Figure 7F).

These results suggest that stigmasterol suppresses senescence‐associated phenotypes, at least in part through downregulation of PAK4.

### 3.8 Stigmasterol Reduces Lesion Aggressiveness and Immune Remodeling In Vivo

**FIGURE 8:**
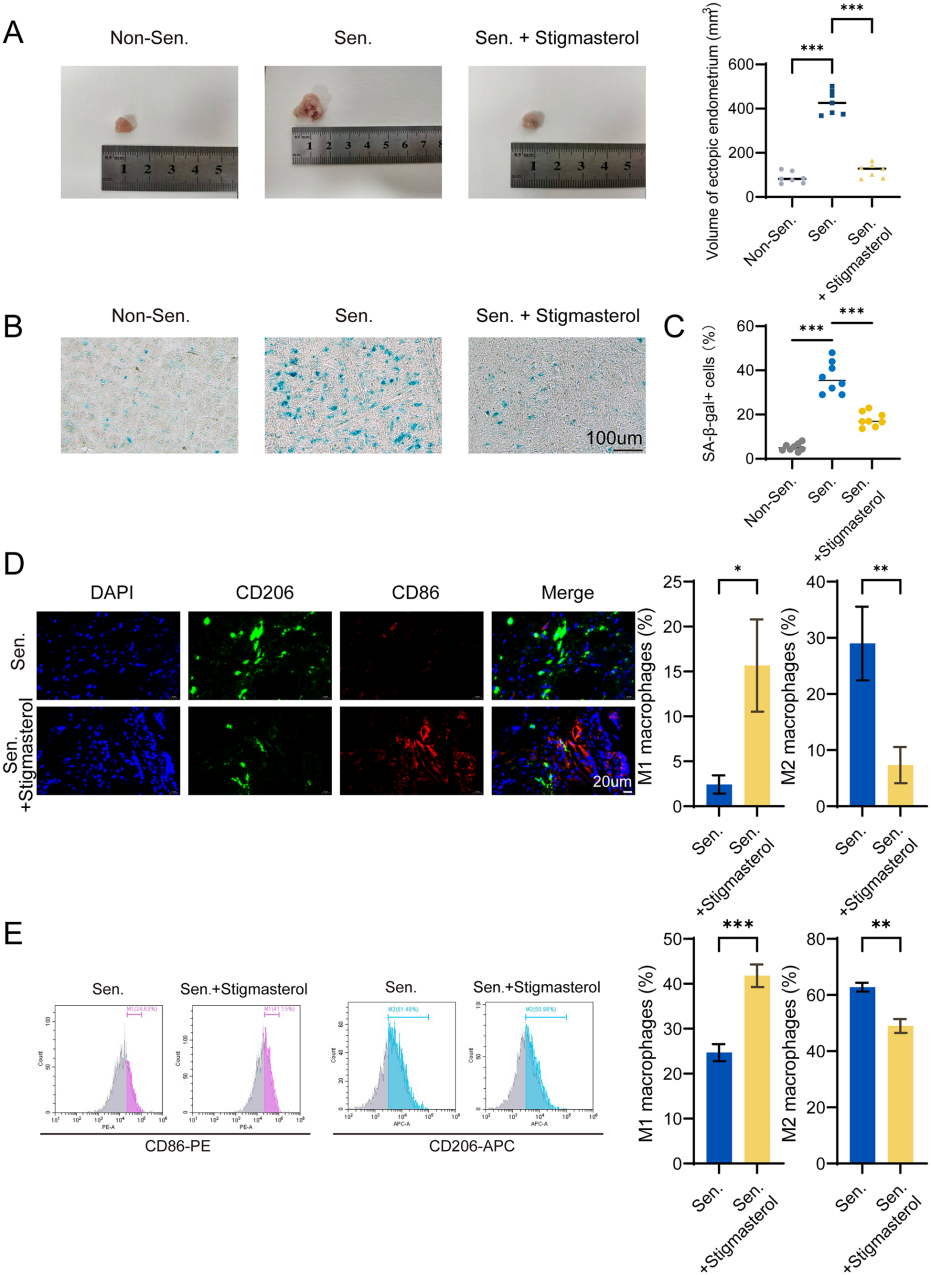
Stigmasterol attenuates lesion growth and M2 macrophage infiltration in vivo. (A) Endometriosis lesion volume was reduced by stigmasterol in a senescent mouse model. (B) SA‐β‐gal staining showed decreased cellular senescence. (C) Quantification of SA‐β‐gal staining confirmed reduced senescence in lesions. (D) Immunofluorescence revealed reduced CD206^+^ and increased CD86^+^ macrophage infiltration. (E) Flow cytometry indicated decreased splenic M2 polarization. *, *p* < 0.05; **, *p* < 0.01; ***, *p* < 0.001; ns, not significant.

Following the in vitro findings, we evaluated the therapeutic effects of stigmasterol in a mouse model of ectopic endometriosis. Mice were assigned to control, senescence (D‐galactose), and senescence + stigmasterol treatment groups. Compared with controls, ectopic lesion volume was significantly increased in the senescence group, whereas stigmasterol treatment markedly reduced lesion size (Figure 8A). SA‐β‐gal staining demonstrated that stigmasterol significantly suppressed senescence within ectopic lesions (Figure 8B,C). Immunofluorescence analysis showed that senescent lesions were enriched in CD206^+^ macrophages and exhibited reduced CD86^+^ macrophages. Stigmasterol treatment markedly decreased M2 macrophage infiltration in ectopic lesions (Figure 8D). Consistently, flow cytometric analysis of splenic macrophages showed that stigmasterol significantly attenuated senescence‐induced M2 polarization at the systemic level (Figure 8E).

Collectively, these results demonstrate that stigmasterol suppresses lesion progression and attenuates senescence‐associated immune remodeling in vivo, supporting its potential therapeutic value in endometriosis.

## 4 Discussion

Ectopic endometrial tissues display several cancer‐like behaviors, including rapid proliferation under hypoxia, extracellular matrix remodeling, enhanced invasiveness, and neovascularization (Artemova et al. 2021). Recent studies have identified strong molecular and clinical correlations between endometriosis and various malignancies, such as endometrial, ovarian, and thyroid cancers (Bulun et al. 2019; Kvaskoff et al. 2021; Terzic et al. 2021). Notably, oncogenic mutations frequently detected in ectopic endometrium parallel those found in tumor tissues, suggesting that such alterations may predispose ectopic lesions to malignant transformation (Lac et al. 2019; Guo 2020). Moreover, the stromal and immune microenvironments of ectopic endometrial lesions mirror those of tumors, further reinforcing the concept of endometriosis as a benign yet tumor‐like disease.

While senescence is classically regarded as a tumor‐suppressive mechanism, accumulating evidence suggests that it can paradoxically promote disease malignancy through the SASP. SASP factors are increasingly recognized as critical regulators of tissue remodeling and immune modulation, capable of establishing chronic inflammatory microenvironments and promoting immune evasion in cancer and fibrotic diseases (Campisi and D'Adda 2007; Salminen 2026). This dualistic role of senescence is increasingly recognized across diverse pathological contexts, including endometriosis, where senescence‐associated changes may similarly drive immune evasion and lesion progression. Previous studies have suggested that senescence escape mechanisms, such as SIRT1 activation, facilitate epithelial‐mesenchymal transition and lesion progression (Wang, Wu, et al. 2022; Wang, Zhang, et al. 2022; Wang et al. 2023). Consistently, our findings reveal a relative deficiency of senescence signatures in ectopic versus eutopic endometrium. Intriguingly, among ectopic lesions, subsets enriched for senescent cells displayed greater invasiveness, resembling the early tumorigenic shift where senescent cell accumulation paradoxically enhances malignancy and therapy resistance.

The progression of endometriosis is intricately linked to its immune microenvironment (Vallve‐Juanico et al. 2019). Macrophages, especially M2‐polarized subsets, dominate the peritoneal cavity of endometriosis patients and contribute to lesion maintenance (Bacci et al. 2009; Xiaocui et al. 2022; Gou et al. 2023). Our study further demonstrates that senescent ectopic lesions are associated with enhanced M2 macrophage infiltration, paralleling findings in solid tumors where senescent cells orchestrate immunosuppressive niches to promote cancer aggressiveness. Consistent with the SASP framework, we observed increased secretion of IL‐1α, IL‐1β, IL‐6, and IL‐8 in senescent ectopic endometrial cells, and IL‐6 neutralization partially attenuated macrophage M2 polarization. These findings support a direct role of SASP‐derived cytokines in shaping the immune microenvironment of endometriotic lesions. This immunological remodeling may diminish phagocytic clearance of ectopic cells and foster a pro‐survival microenvironment.

Recent studies have suggested that cellular senescence contributes to persistent inflammatory and oxidative stress states in endometriotic tissues. Senescence‐associated alterations have been linked to increased reactive oxygen species production and enhanced inflammatory signaling in ectopic endometrium, which may favor long‐term lesion persistence and tissue remodeling (Malvezzi, Dobo, Filippi, Mendes Do Nascimento, et al. 2022; Malvezzi, Dobo, Filippi, Mendes, et al. 2022; Malvezzi et al. 2023; Ochoa et al. 2025). Although oxidative stress was not directly assessed in the present study, the enhanced invasiveness and immune remodeling observed in senescence‐enriched lesions support the concept that senescence may contribute to the chronic and progressive nature of endometriosis.

In our study, a PAK4‐centered senescence signature identified specific subtypes of ectopic endometrial cells. PAK4 has previously been implicated in promoting the activity and invasiveness of ectopic endometrial cells, as well as lesion progression, by activating pathways such as PI3K/AKT and ERK/MEK (Kim et al. 2013). Additionally, PAK4 is responsive to cellular senescence signals and, under certain conditions, can promote premature senescence through a positive feedback mechanism (Cammarano et al. 2005). Our data reveal that high PAK4 expression not only enhances the invasiveness of ectopic endometrial cells but also induces a senescence‐like phenotype via activation of the PI3K/AKT pathway. This pathological senescence is accompanied by increased local infiltration of M2‐polarized macrophages, contributing to an immunosuppressive microenvironment that further accelerates lesion dissemination and deterioration. This PAK4‐driven senescence‐immune interaction may shape a tissue microenvironment conducive to sustained survival and invasion of ectopic lesions.

Interestingly, prior studies have highlighted the context‐dependent nature of PAK4, with distinct roles in senescence regulation across different tissue types (Costa et al. 2019). In malignant tissues, PAK4 activity is typically associated with proliferative signaling and therapy resistance, whereas our findings indicate that in ectopic endometrial tissues PAK4 preferentially promotes a senescence‐associated remodeling program. One possible explanation is that disease‐specific signaling thresholds may determine the biological outcome of PAK4 activation. Moderate activation of PI3K/AKT signaling in benign ectopic tissues may favor senescence‐associated remodeling and SASP production, whereas stronger or sustained activation in malignant contexts may drive uncontrolled proliferation. This context‐dependent behavior highlights the importance of tissue‐specific signaling environments in shaping PAK4‐mediated cellular responses.

SOCS1, HMGA1, and NCAPH2 are three additional signature genes that are overexpressed in senescent ectopic endometrial tissues. SOCS1 has been reported to regulate macrophage M1 polarization by inhibiting the JAK2/STAT1 pathway in various cell types and is involved in the p53‐dependent cellular senescence process (Calabrese et al. 2009; Beaurivage et al. 2016; Chang et al. 2020; Zhao et al. 2023). HMGA1, a classical senescence marker, is typically upregulated in highly proliferative cells and confers resistance and stemness characteristics (Li et al. 2021; Sgubin et al. 2022; Wang, Wu, et al. 2022; Wang, Zhang, et al. 2022; Yang et al. 2024). NCAPH2 participates in the regulation of senescence‐associated chromatin alterations by maintaining telomere stability (Wallace et al. 2019). Overall, these signature genes reflect a multifaceted alteration of the ectopic microenvironment.

Stigmasterol, a natural phytosterol, has been shown to exert anti‐inflammatory, antioxidative, and anti‐proliferative effects by modulating signaling pathways such as PI3K/AKT, NF‐κB, and JAK/STAT (Ahmad et al. 2020; Haque et al. 2021; Zhao et al. 2021; Jie et al. 2022; Mongkolpobsin et al. 2023). In the context of endometriosis, our study demonstrated that stigmasterol effectively suppressed PAK4 expression, attenuated senescence‐associated phenotypes, and reduced M2 macrophage infiltration. These findings suggest that stigmasterol may disrupt the pathological interplay between senescent cells and the immunosuppressive microenvironment, thereby impeding lesion progression. Nevertheless, challenges such as poor solubility and low bioavailability may constrain its therapeutic application, highlighting the need for formulation optimization or structural modification in future studies (Feng et al. 2021).

Several limitations should be acknowledged in this study. First, although we established a robust senescence scoring model and validated its association with invasive and immunosuppressive phenotypes, the direct validation of senescence dynamics in human ectopic tissues was limited by sample availability. It should be noted that the senescence score proposed in this study is currently intended for research‐based biological stratification and mechanistic exploration, rather than immediate clinical decision‐making. Future validation in larger prospective cohorts will be required before potential clinical application. Second, while we demonstrated that PAK4 promotes senescence and immune remodeling, the downstream signaling cascades linking PAK4 activation to M2 macrophage polarization remain to be fully elucidated. Third, although stigmasterol demonstrated therapeutic efficacy in vitro and in preclinical models, its pharmacokinetic limitations, including poor solubility and bioavailability, may restrict immediate clinical translation. Future studies will be required to optimize its pharmacological properties, including improved formulation strategies and delivery approaches, in order to enhance its translational potential.

Future studies should aim to comprehensively dissect the molecular mechanisms by which PAK4 orchestrates senescence and immune microenvironment remodeling, possibly involving cross‐talk with pathways such as PI3K/Akt, STAT3, or TGF‐β signaling. Additionally, expanding the evaluation of stigmasterol to humanized models or patient‐derived xenografts would provide stronger translational evidence. Formulation strategies, such as nanoparticle‐based delivery systems, may enhance stigmasterol's therapeutic potential. Ultimately, targeting senescence‐driven immune modulation represents a promising avenue not only for controlling lesion progression in endometriosis but also for mitigating recurrence and improving patient outcomes.

## 5 Conclusions

In conclusion, our study reveals that ectopic endometrial lesions are characterized by reduced senescence enrichment yet display significant heterogeneity, with senescence‐high subsets exhibiting enhanced invasiveness and immunosuppressive remodeling. Through the construction of a six‐gene senescence scoring model, we identified PAK4 as a dominant regulator linking cellular senescence to immune microenvironment reprogramming. Functional experiments demonstrated that PAK4 promotes senescence‐associated phenotypes and M2 macrophage polarization, thereby driving lesion progression. Importantly, stigmasterol effectively suppressed PAK4 expression, mitigated pathological senescence, and reduced M2 infiltration, highlighting its potential as a therapeutic agent targeting senescence‐driven pathologies in endometriosis. These findings provide new mechanistic insights into endometriosis progression and propose a novel therapeutic strategy centered on senescence and immune modulation.

## Author Contributions

**Jingchun Liu:** writing – review and editing, writing – original draft, visualization, supervision, software, methodology, investigation, data curation, funding acquisition. **Wuyue Han:** methodology, investigation, data curation. **Jianming Tang:** visualization, software. **Huanzhi Wan:** methodology, funding acquisition. **Haoyu Wang:** visualization, software. **Jiaxin Peng:** visualization, software. **Wenjing Ma:** visualization, software. **Min Hu:** visualization, software. **Li Hong:** writing – review and editing, writing – original draft, funding acquisition.

## Funding

This research was funded by the Open Project of Hubei Provincial Key Laboratory (2024KFZ015).

## Ethics Statement

The study was approved by the Ethical Review Committee of Renmin Hospital of Wuhan University in accordance with the principles in the Basel Declaration (NO. WDRM‐20230410D).

## Consent

All authors have acknowledged the manuscript and agreed to its publication.

## Conflicts of Interest

The authors declare no conflicts of interest.

## Supporting information


**Figure S1:** Senescence characteristics show differences in ectopic endometrium.
**Figure S2:** Senescence promotes the expansion of ectopic endometrial lesions.
**Figure S3:** Highly senescent ectopic endometrium reflects higher M2 macrophage infiltration.
**Figure S4:** Immune landscape and M2‐type macrophage ratios.
**Figure S5:** Neutralization of IL‐6 in MEECs‐ and MESCs‐derived CM partially attenuates macrophage M2 polarization.
**Figure S6:** PAK4 knockdown suppresses the invasive capacity of senescent ectopic endometrial cells.
**Figure S7:** Knockdown of additional senescence signature genes shows limited effects on cellular senescence in ectopic endometrial cells.
**Figure S8:** Convergent enrichment of PI3K/AKT signaling in senescent and PAK4‐high ectopic endometrium.

**Table S1:** Primer sequences.

**Table S2:** 217 genes associated with senescence validated in non‐tumor cells.

**Table S3:** Molecular docking parameters.

## Data Availability

Data will be made available on request.
