# Correction to “Senescence‐Driven Remodeling Defines an Aggressive and Immunomodulatory Subtype of Endometriosis”

**DOI:** 10.1111/acel.70584

**Published:** 2026-06-10

Liu, J., W. Han, J. Tang, et al. 2026. Senescence‐Driven Remodeling Defines an Aggressive and Immunomodulatory Subtype of Endometriosis. *Aging Cell* 25, no. 4: e70463. https://doi.org/10.1111/acel.70463.

In Figure 6E, the representative image for the senescence‐treated MEEC group was inadvertently replaced by the image corresponding to the senescence‐treated MESC group shown in Figure 7D during figure assembly. The corrected Figure 6 is shown below.

**FIGURE 6:**
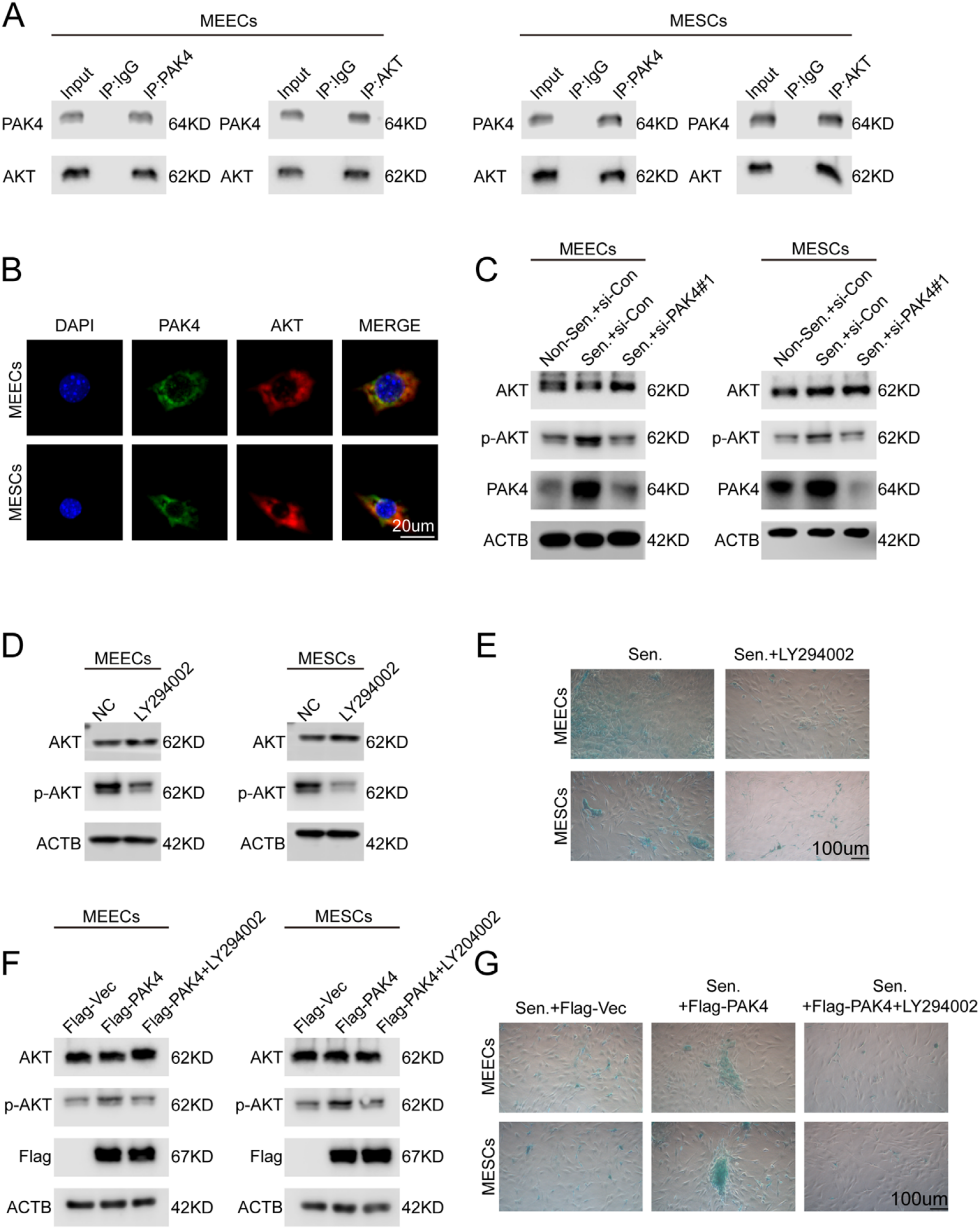
PAK4 promotes cellular senescence through activation of the PI3K/AKT signaling pathway. (A) Co‐IP analysis showing interaction between endogenous PAK4 and AKT in MEECs and MESCs. (B) Immunofluorescence images demonstrating colocalization of PAK4 and AKT in MEECs and MESCs. (C) Western blot showing decreased AKT phosphorylation following PAK4 knockdown under D‐gal‐induced senescence conditions. (D, E) Western blot showing that LY294002 treatment inhibits AKT phosphorylation. (E) SA‐β‐gal staining indicating that LY294002 reduces cellular senescence in MEECs and MESCs. (F) Western blot showing that LY294002 blocks AKT phosphorylation even in the presence of PAK4 overexpression. (G) SA‐β‐gal staining showing that LY294002 reverses PAK4 overexpression‐induced senescence. *, *p* < 0.05; **, *p* < 0.01; ***, *p* < 0.001; ns, not significant.

All other parts of the article remain intact, valid, and unchanged. These corrections do not affect the quantitative analyses, statistical results, data interpretation, or conclusions of the article.

We apologize for this error.

