# Supplementary material for: Senescence‐Driven Remodeling Defines an Aggressive and Immunomodulatory Subtype of Endometriosis
Source: Aging Cell. 2026 Mar 27;25(4):e70463. doi: 10.1111/acel.70463 (PMC13140525; doi:10.1111/acel.70463)
Supplement: Supplementary file 1 — Figure S1: Senescence characteristics show differences in ectopic endometrium. Figure S2: Senescence promotes the expansion of ectopic endometrial lesions. Figure S3: Highly senescent ectopic endometrium reflects higher M2 macrophage infiltration. Figure S4: Immune landscape and M2‐type macrophage ratios. Figure S5: Neutralization of IL‐6 in MEECs‐ and MESCs‐derived CM partially attenuates macrophage M2 polarization. Figure S6: PAK4 knockdown suppresses the invasive capacity of senescent ectopic endometrial cells. Figure S7: Knockdown of additional senescence signature genes shows limited effects on cellular senescence in ectopic endometrial cells. Figure S8: Convergent enrichment of PI3K/AKT signaling in senescent and PAK4‐high ectopic endometrium. [file ACEL-25-e70463-s002.docx]

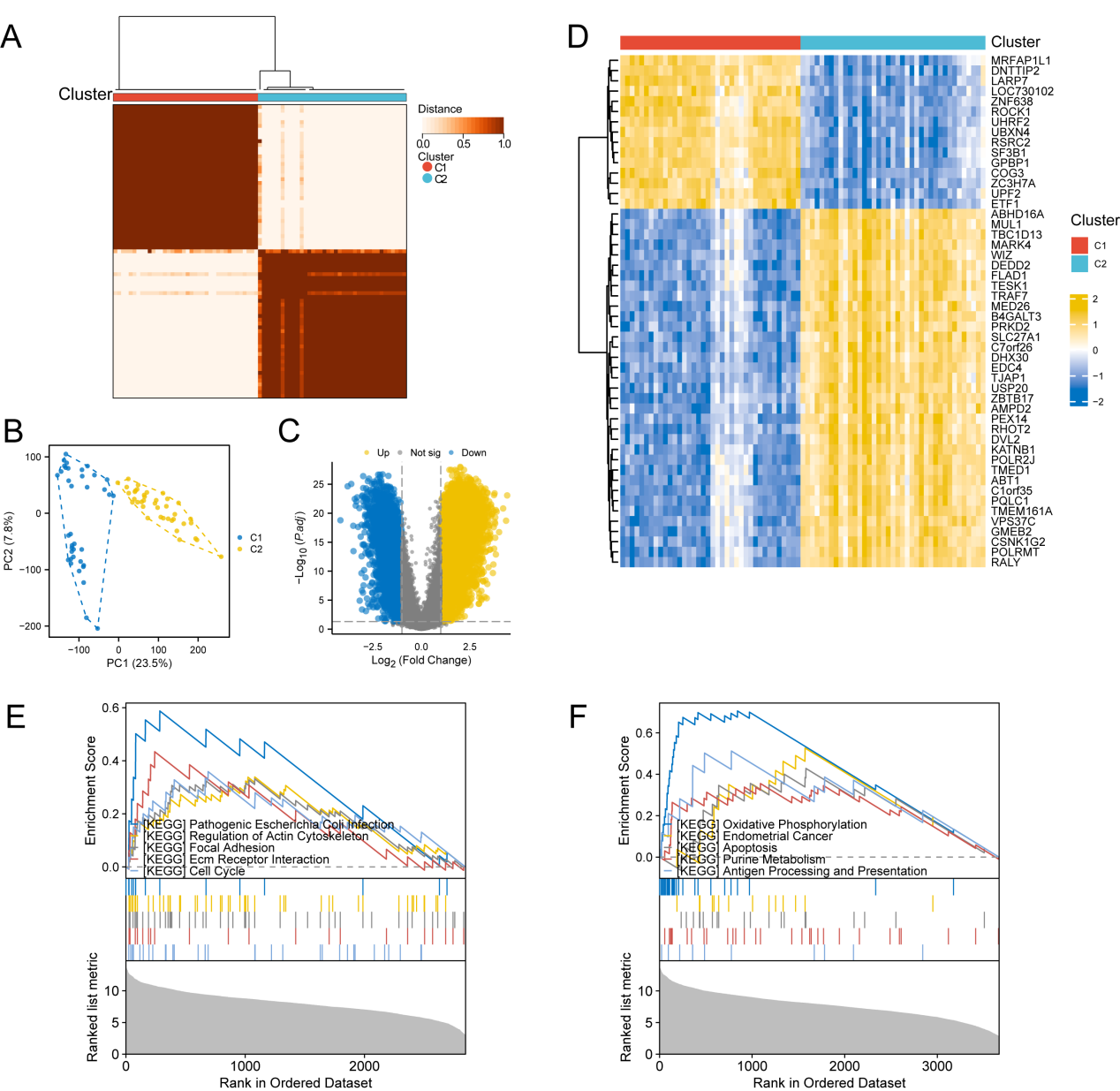


**Supplementary Fig. 1 Senescence characteristics show differences in ectopic endometrium.**

1. Cluster analysis of senescence-related differential genes in the GSE51981 dataset. The optimal clustering is shown when K=2. (B) PCA clustering of two clustered samples. (C) Volcano map of differential genes between cluster 1 and cluster 2. (D) The top 50 most representative differentially expressed genes and their expression levels in both groups. (E-F) GSEA enrichment analysis of (E) down-regulated and (F) up-regulated genes in cluster 2.

**
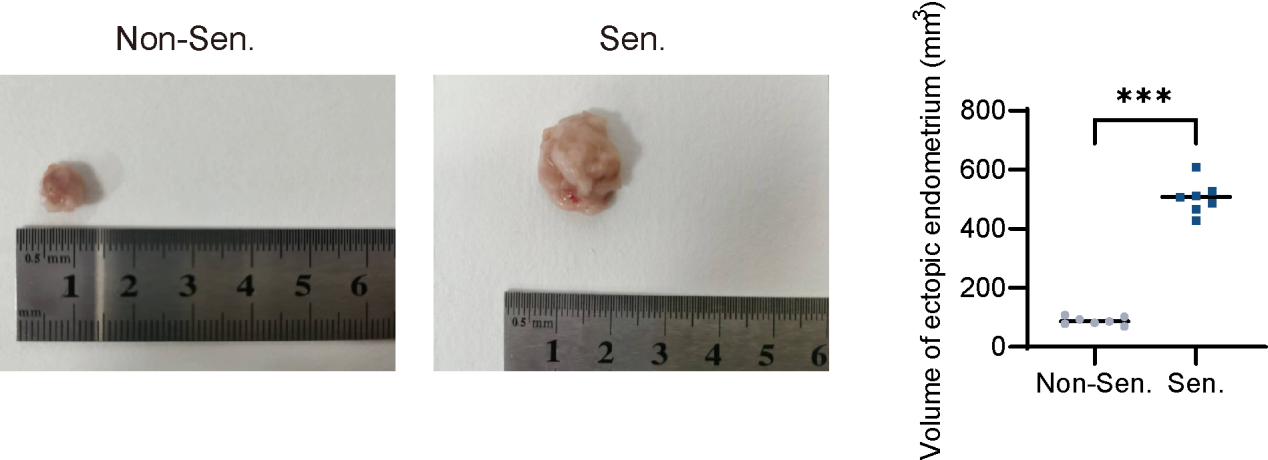
**

**Supplementary Fig. 2 Senescence promotes the expansion of ectopic endometrial lesions.**

A senescence model was established using β-gal staining, followed by collection of ectopic endometrial lesions and measurement of lesion volume. ***, p < 0.001.


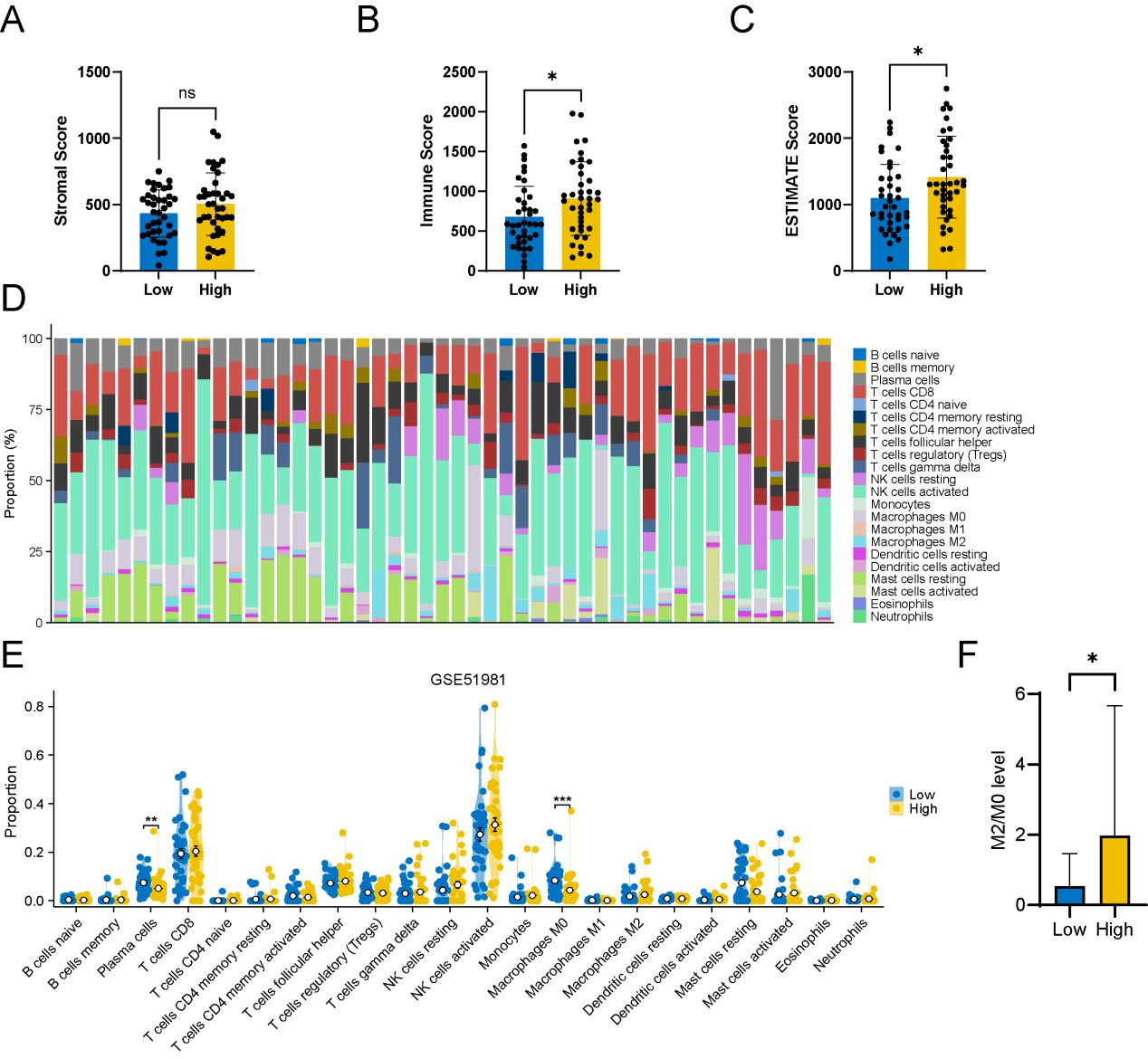


**Supplementary Fig. 3 Highly senescent ectopic endometrium reflects higher M2 macrophage infiltration.**

(A-C) Calculation of (A) stromal score, (B) immune score and (C) ESTIATE score based on ESTIATE algorithm for non-senescent and senescent ectopic endometrium in GSE51981 dataset. (D) Immunoscape of ectopic endometrium in the GSE51981 dataset based on the Cibersort algorithm. (E) Proportion of 22 immune cells in non-senescent and senescent ectopic endometrium in the GSE51981 dataset. (F) Relative levels of M2 macrophages in non-senescent and senescent ectopic endometrium in the GSE51981 dataset. *, p<0.05; **, p<0.01; ***, p<0.001; ns, p≥0.05.


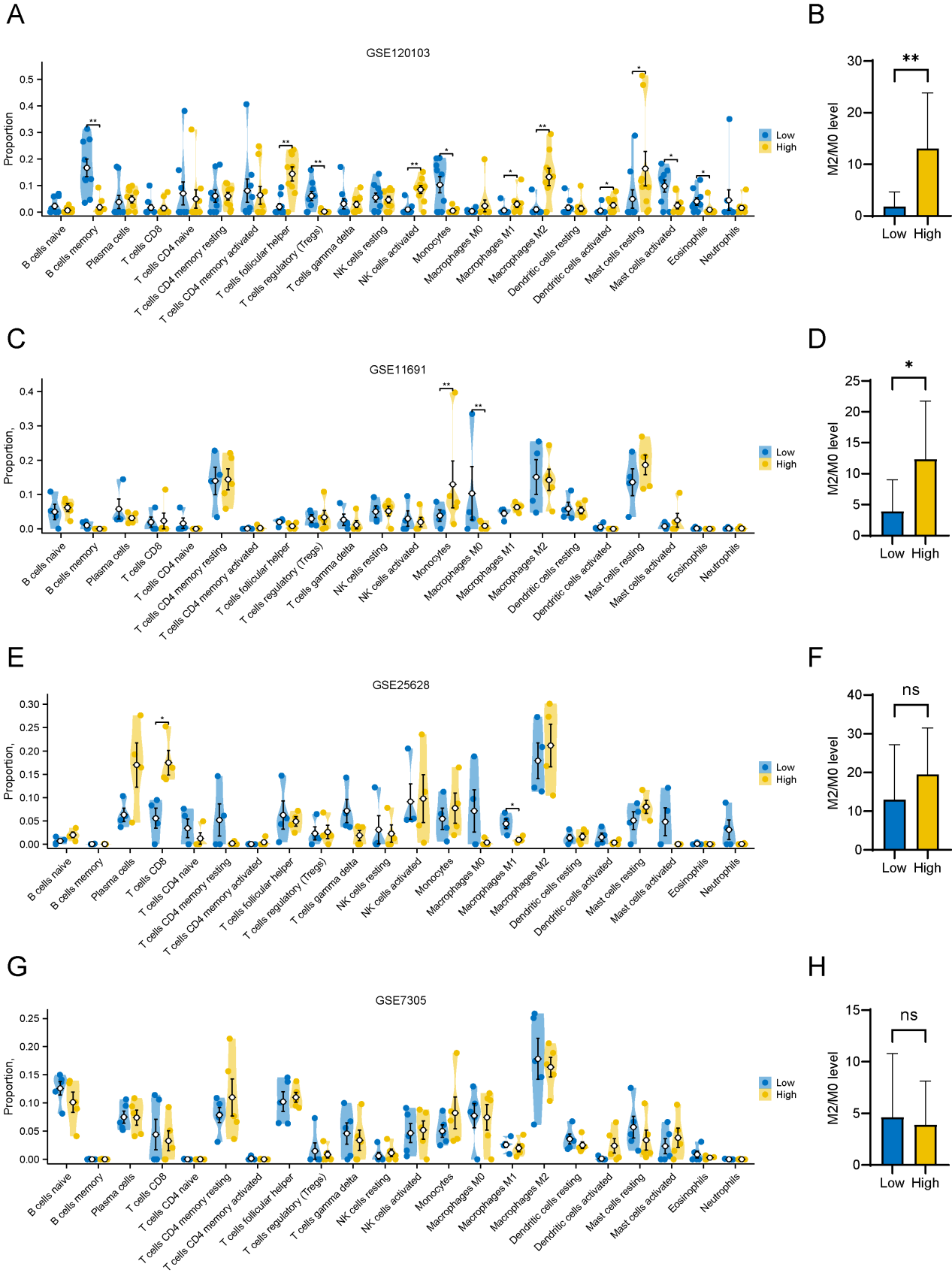


**Supplementary Fig. 4 Immune landscape and M2-type macrophage ratios.**

(A-B) Relative proportions of 22 immune cell types (A) and M2 macrophages (B) in non-senescent vs. senescent ectopic endometrial tissues from the GSE120103 dataset. (C-D) Relative proportions of 22 immune cell types (C) and M2 macrophages (D) in the GSE11691 dataset. (E-F) Immune cell composition (E) and M2 macrophage ratios (F) in the GSE25628 dataset. (G-H) Immune cell composition (G) and M2 macrophage ratios (H) in the GSE7305 dataset. *, p < 0.05; **, p < 0.01; ns, not significant.


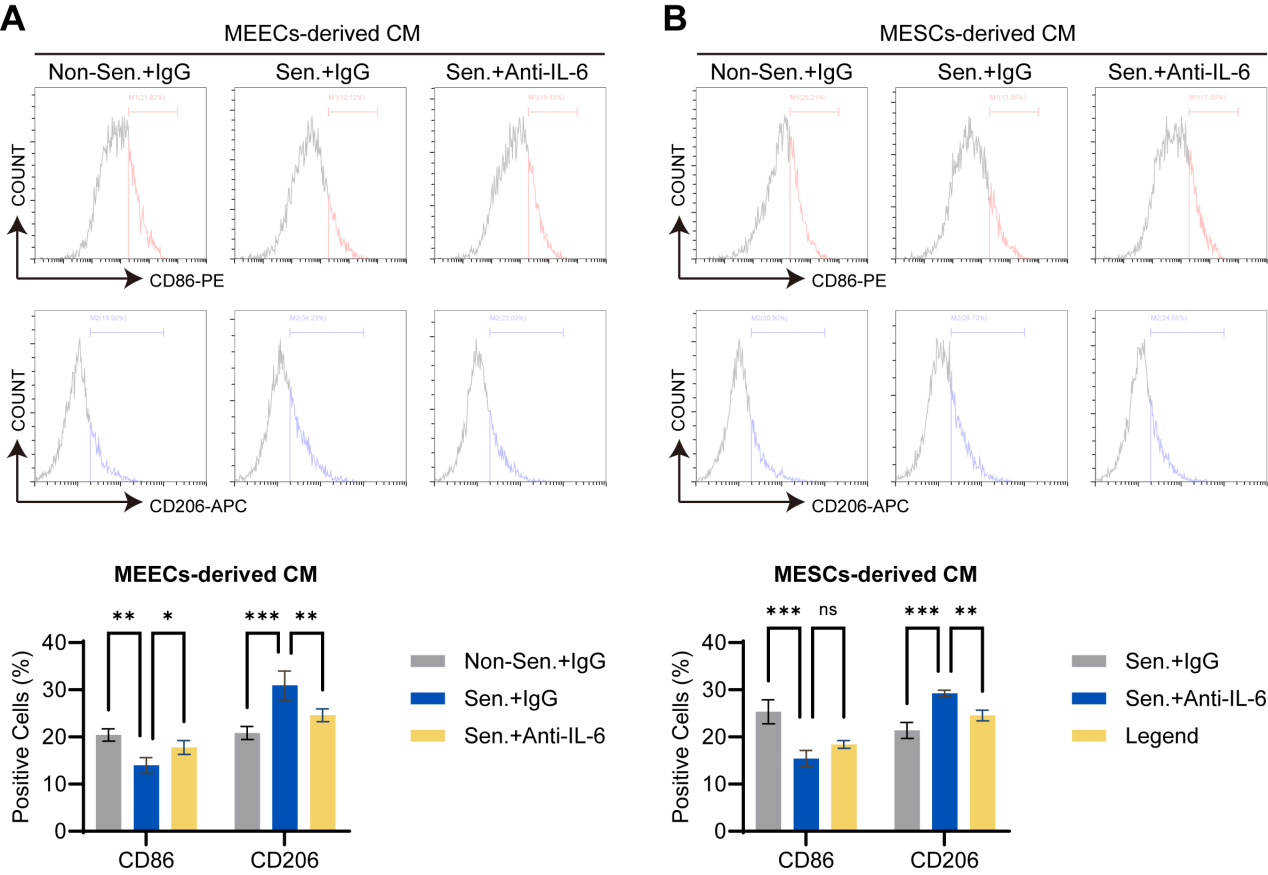


**Supplementary Fig. 5 Neutralization of IL-6 in MEECs- and MESCs-derived CM partially attenuates macrophage M2 polarization.**

(A-B) RAW264.7 macrophages were treated with CM derived from non-senescent or senescent MEECs(A) and MESCs(B) in the presence of isotype IgG or neutralizing anti-IL-6 antibody. *, p < 0.05; **, p < 0.01; ***, p < 0.001; ns, not significant.


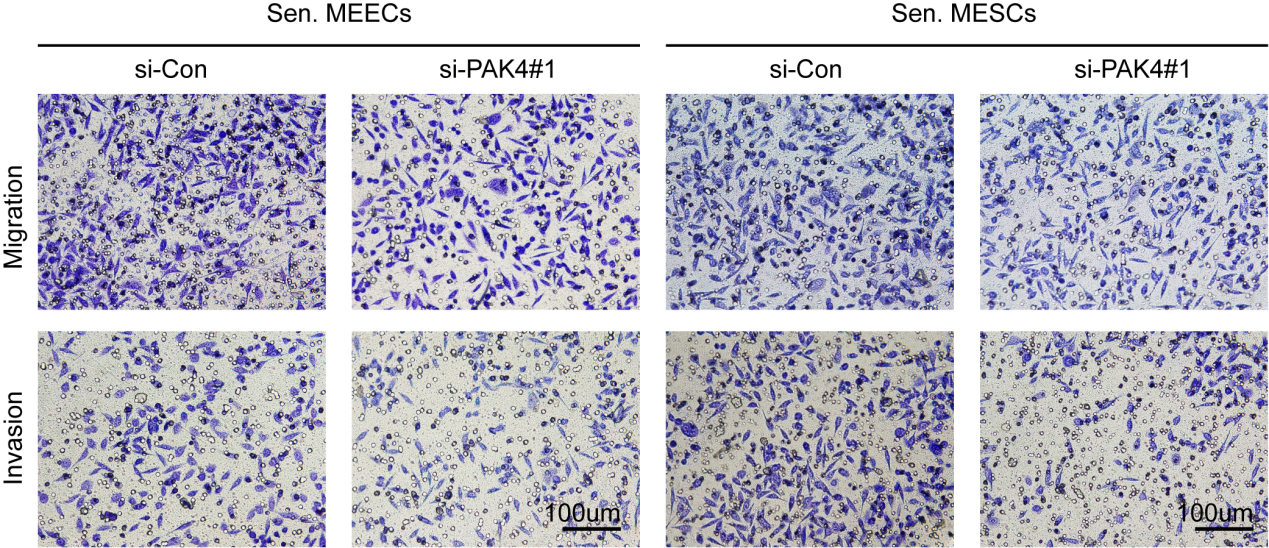


**Supplementary Fig. 6 PAK4 knockdown suppresses the invasive capacity of senescent ectopic endometrial cells.**

Representative images of Transwell migration and invasion assays in senescent MEECs and MESCs after PAK4 knockdown.


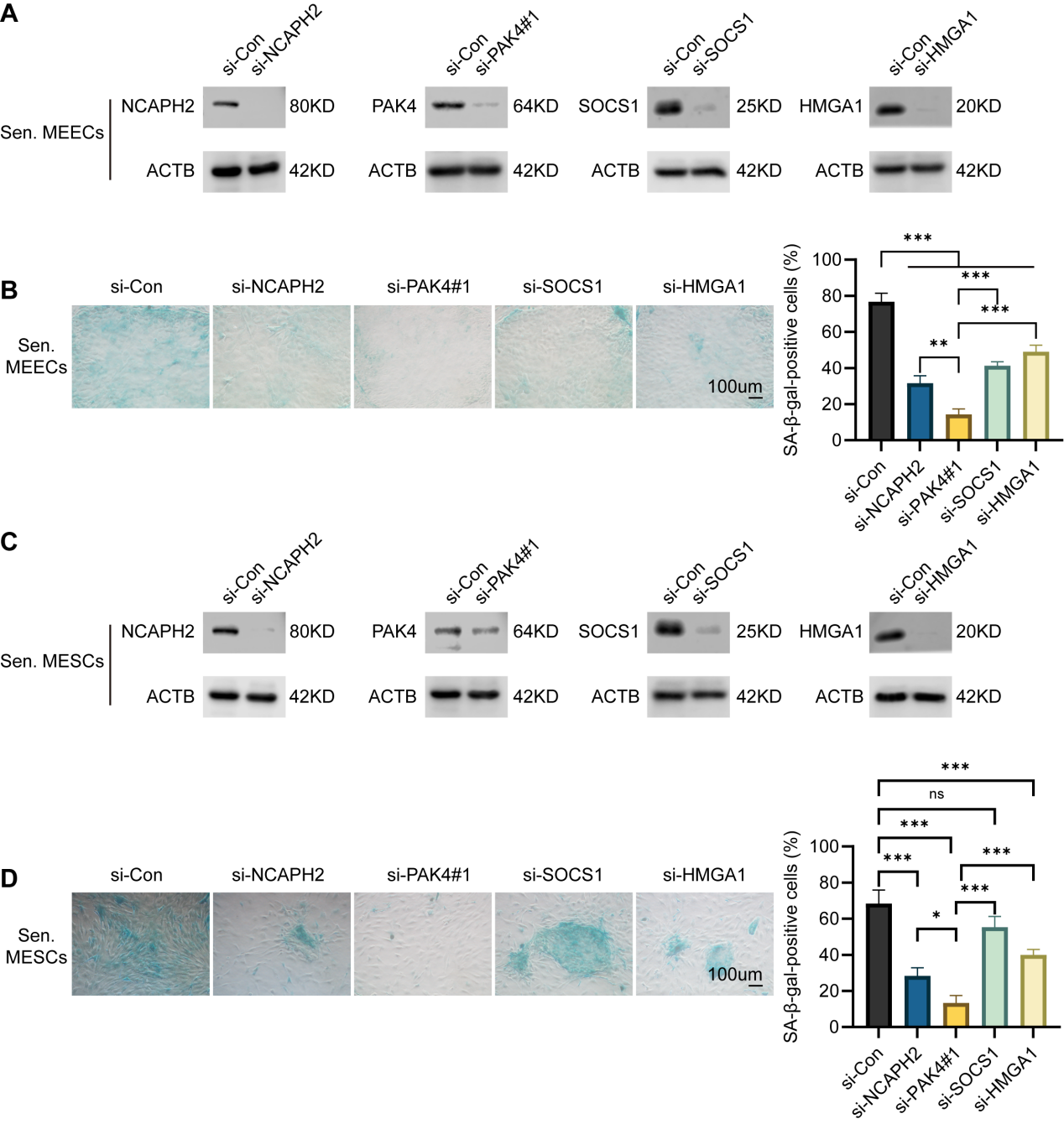


**Supplementary Fig. 7 Knockdown of additional senescence signature genes shows limited effects on cellular senescence in ectopic endometrial cells.**

(A) Knockdown efficiency of NCAPH2, PAK4, SOCS1, and HMGA1 in MEECs was confirmed by western blot. (B) Representative images of SA-β-gal staining in MEECs following siRNA-mediated knockdown of the indicated genes. (C) Knockdown efficiency of NCAPH2, PAK4, SOCS1, and HMGA1 in MESCs. (D) Representative images of SA-β-gal staining in MESCs after knockdown of the indicated genes. *, p < 0.05; **, p < 0.01; ***, p < 0.001; ns, not significant.


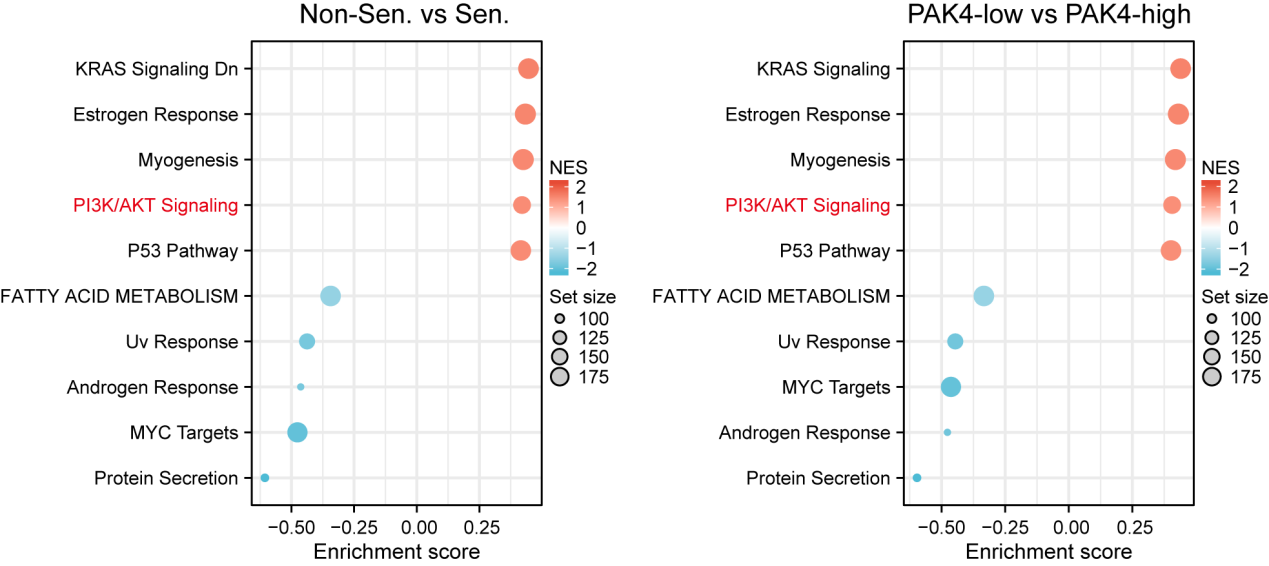


**Supplementary Fig. 8 Convergent enrichment of PI3K/AKT signaling in senescent and PAK4-high ectopic endometrium.**

GSEA comparing non-senescent vs senescent ectopic endometrial, and PAK4-low vs PAK4-high samples in the GSE51981 dataset. PI3K/AKT is one of the overlapping pathways.
