# Supplementary material for: Senescence‐Driven Remodeling Defines an Aggressive and Immunomodulatory Subtype of Endometriosis
Source: Aging Cell. 2026 Mar 27;25(4):e70463. doi: 10.1111/acel.70463 (PMC13140525; doi:10.1111/acel.70463)
Supplement: Supplementary file 2 — Table S1: Primer sequences. [file ACEL-25-e70463-s001.pdf]

Supplementary Table 1. Primer sequences.

Gene	FORWARD	REVERSE
Brd7	TGATCTGTCTCCTGGGTCCTTCTTAC	TGGTCTCCTTAGGTTCTTCACACTCTC
Socs1	TCGCCAACGGAACTGCTTCTTC	TGGAAGGGGAAGGAACTCAGGTAG
Ogt	TAGACTGCCTTCTGTCCATCCTCAC	GCTTCCGCCATCACCTTCACTC
Hmga1	CAAGCAGGAAAAGGATGGGACTGAG	GCCCTCCTCTTCCTCCTTCTCC
Ncaph2	GATGCCTATGCTGAACACCCTGAC	TCTTCCAAGCGATCCTCCTCTGTAG
Pak4	CACACCAGGATGAACGAGGAACAG	CCATAGGGAAGGCGGGAGATGAG
