# Supplementary material for: Senescence‐Driven Remodeling Defines an Aggressive and Immunomodulatory Subtype of Endometriosis
Source: Aging Cell. 2026 Mar 27;25(4):e70463. doi: 10.1111/acel.70463 (PMC13140525; doi:10.1111/acel.70463)
Supplement: Supplementary file 3 — Table S2: 217 genes associated with senescence validated in non‐tumor cells. [file ACEL-25-e70463-s003.pdf]

Supplementary Table 2. 217 genes associated with senescence validated in non-tumor cells.

Gene Symbol	Entrez Id	Method	Cell Types	Cell Lines	Cancer Line?	Senescence Type	Senescence Effect
AAK1	22848	Overexpression	Embryonic kidney, Lung fibroblast	HEK293T, IMR-90, MRC-5	No	Unclear	Induces
ACKR1	2532	Overexpression	Cerebral-microvascular endothelial	HCEC	No	Stress-induced	Induces
ADCK5	203054	Overexpression	Embryonic kidney, Lung fibroblast	HEK293T, IMR-90, MRC-5	No	Unclear	Induces
AGT	183	Knockout, Overexpression	Vascular smooth muscle	Primary cell	No	Stress-induced	Induces
AHR	196	Knockdown	Embryonic kidney	HEK	No	Stress-induced	Induces
AKT1	207	Overexpression	Aortic endothelial, Dermal microvascular endothelial, Umbilical vein endothelial	Primary cell	No	Oncogene-induced	Induces
ALOX15B	247	Overexpression	Prostate epithelial	Primary cell	No	Unclear	Induces
AR	367	Knockdown, Overexpression	Hair follicular keratinocyte	Primary cell	No	Unclear	Induces
ARF1	375	Knockdown	Lung fibroblast	IMR-90	No	Oncogene-induced	Induces
ARG2	384	Mutation, Overexpression	Umbilical vein endothelial, Vascular smooth muscle	HUVEC, VSMC	No	Unclear	Induces
ARID1A	8289	Knockdown	Pancreas epithelial-like	HPNE	No	Oncogene-induced	Induces
ARID1B	57492	Knockdown	Lung fibroblast	IMR-90	No	Oncogene-induced	Induces
ASF1A	25842	Knockout, Overexpression	Lung fibroblast	Primary cell	No	Oncogene-induced, Replicative	Induces
ASXL2	55252	Mutation, Overexpression	Lung fibroblast	IMR-90	No	Unclear	Induces
ATF6	22926	Knockdown, Mutation	Breast cancer, Foreskin fibroblast	MCF-7, NHDF	No	Oncogene-induced, Replicative, Stress-induced	Induces
ATXN10	25814	Knockdown	Mammary fibroblast	HMF3A	No	Unclear	Induces
AXL	558	Overexpression	Embryonic kidney, Lung fibroblast	HEK293T, IMR-90, MRC-5	No	Unclear	Induces
BAP1	8314	Mutation, Overexpression	Lung fibroblast	IMR-90	No	Unclear	Induces
BCL2	596	Overexpression	Lung fibroblast	IMR-90	No	Oncogene-induced	Induces
BIN1	274	Knockdown	Foreskin fibroblast, Melanocyte	BJ, Primary cell	No	Oncogene-induced	Induces
BLK	640	Overexpression	Embryonic kidney, Lung fibroblast	HEK293T, IMR-90, MRC-5	No	Unclear	Induces
BNIP3L	665	Knockdown	Foreskin fibroblast, Melanocyte	BJ, Primary cell	No	Oncogene-induced	Induces
BRAF	673	Overexpression	Foreskin fibroblast, Melanocyte	BJ, Primary cell	No	Oncogene-induced	Induces
BRD7	29117	Knockdown	Foreskin fibroblast	BJ	No	Replicative	Induces
BTG2	7832	Knockdown, Overexpression	Foreskin fibroblast	BJ	No	Replicative	Induces
CARF	79800	Overexpression	Lung fibroblast	MRC-5	No	Replicative, Stress-induced	Induces
CASP2	835	Knockdown	Mammary epithelial	Primary cell	No	Oncogene-induced	Induces
CAV1	857	Knockdown	Fibroblast	Primary cell	No	Unclear	Induces
CCL2	6347	Knockdown	Mesenchymal stem	Primary cell	No	Unclear	Induces
CCND1	595	Overexpression	Fibroblast	HS68, WI-38	No	Unclear	Induces
CD34	947	Mutation	Dental pulp stem	Primary cell	No	Unclear	Induces
CDK18	5129	Overexpression	Embryonic kidney, Lung fibroblast	HEK293T, IMR-90, MRC-5	No	Unclear	Induces
CDKN1A	1026	Knockout	Fibroblast	LF1	No	Oncogene-induced, Replicative, Stress-induced	Induces
CDKN1B	1027	Overexpression	Lung fibroblast	TIG-3	No	Unclear	Induces
CDKN2A	1029	Overexpression	Lung fibroblast	TIG-3	No	Oncogene-induced, Replicative, Stress-induced	Induces
CDKN2AIP	55602	Overexpression	Lung fibroblast	MRC-5, TIG-1	No	Replicative, Stress-induced	Induces
CEACAM1	634	Knockdown	Mammary epithelial	MCF-10A	No	Stress-induced	Induces
CHD5	26038	Knockdown	Lung fibroblast	IMR-90	No	Oncogene-induced, Replicative	Induces
CKB	1152	Overexpression	Embryonic kidney, Lung fibroblast	HEK293T, IMR-90, MRC-5	No	Unclear	Induces
CLCA2	9635	Knockdown	Lung fibroblast	IMR-90	No	Stress-induced	Induces
CPEB1	64506	Knockout, Overexpression	Foreskin fibroblast, Lung fibroblast	Primary cell, WI-38	No	Replicative	Induces
CTNNB1	1499	Knockdown	Bone marrow mesenchymal stem	Primary cell	No	Unclear	Induces
CXCL1	2919	Overexpression	Ovarian fibroblast	NOF150	No	Oncogene-induced	Induces
CXCR2	3579	Knockdown, Overexpression	Lung fibroblast, Mammary epithelial	IMR-90, Primary cell, WI-38	No	Oncogene-induced, Replicative	Induces
CYBB	1536	Knockdown	Endodthelial progenitor	Primary cell	No	Unclear	Induces
DDB2	1643	Knockout	Lung fibroblast	IMR-90	No	Oncogene-induced	Induces
DHCR24	1718	Mutation	Lung fibroblast	WI-38	No	Oncogene-induced	Induces
DMTF1	9988	Knockdown	Foreskin fibroblast, Melanocyte	BJ, Primary cell	No	Oncogene-induced	Induces
DPP4	1803	Knockdown, Overexpression	Embryonic kidney, Fibroblast	BJ, HEK293T, TIG-3	No	Oncogene-induced	Induces
E2F1	1869	Overexpression	Lung fibroblast	WI-38	No	Unclear	Induces
E2F7	144455	Knockdown	Foreskin fibroblast, Lung fibroblast	BJ, IMR-90, WI-38	No	Oncogene-induced	Induces
EEF1E1	9521	Knockdown, Overexpression	Umbilical cord blood-derived mesenchymal stem	HUCB	No	Unclear	Induces
EGR2	1959	Knockdown	Mammary epithelial	IMR-90, Primary cell, WI-38	No	Unclear	Induces
EHF	26298	Overexpression	Lung fibroblast	WI-38	No	Unclear	Induces
EIF2AK2	5610	Knockdown	Umbilical vein endothelial	HUVEC	No	Stress-induced	Induces
ENTPD7	57089	Knockdown	Lung fibroblast	IMR-90	No	Oncogene-induced	Induces
EP300	2033	Knockdown	Lung fibroblast	IMR-90	No	Oncogene-induced, Replicative	Induces
ERRFI1	54206	Overexpression	Lung fibroblast	WI-38	No	Unclear	Induces
ERVW-1	30816	Overexpression	Lung fibroblast, Mammary epithelial	IMR-90, MCF-10A	No	Oncogene-induced	Induces
ETS1	2113	Overexpression	Foreskin fibroblast, Lung fibroblast	HS68, TIG-3	No	Oncogene-induced	Induces
ETS2	2114	Overexpression	Foreskin fibroblast, Lung fibroblast	HS68, TIG-3	No	Oncogene-induced	Induces
ETV6	2120	Knockdown	Foreskin fibroblast	NHDF	No	Unclear	Induces
EWSR1	2130	Knockout	Hematopoietic stem	Primary cell	No	Unclear	Induces
FANCD2	2177	Knockdown	Dermal fibroblast	Primary cell	No	Stress-induced	Induces
FASN	2194	Knockdown	Foreskin fibroblast	HFFF2	No	Oncogene-induced	Induces

FASTK	10922	Overexpression	Embryonic kidney, Lung fibroblast	HEK293T, IMR-90, MRC-5	No	Unclear	Induces
FOXO1	2308	Knockdown	Foreskin fibroblast	NHDF	No	Unclear	Induces
GATA4	2626	Knockdown, Overexpression	Foreskin fibroblast, Lung fibroblast	BJ, IMR-90	No	Replicative, Stress-induced	Induces
GDF15	9518	Knockdown, Overexpression	Aortic endothelial	Primary cell	No	Oncogene-induced, Stress-induced	Induces
GJA1	2697	Overexpression	Chondrocyte	T/C-28a2	No	Unclear	Induces
GNG11	2791	Knockout, Overexpression	Lung fibroblast	TIG-7	No	Stress-induced	Induces
GRK4	2868	Overexpression	Embryonic kidney	HEK293	No	Unclear	Induces
GRK6	2870	Overexpression	Embryonic kidney, Lung fibroblast	HEK293T, IMR-90, MRC-5	No	Unclear	Induces
HBP1	26959	Knockdown	Lung fibroblast	WI-38	No	Oncogene-induced	Induces
HIRA	7290	Overexpression	Lung fibroblast	WI-38	No	Oncogene-induced	Induces
HK3	3101	Overexpression	Embryonic kidney, Lung fibroblast	HEK293T, IMR-90, MRC-5	No	Unclear	Induces
HMGA1	3159	Overexpression	Lung fibroblast	IMR-90	No	Oncogene-induced	Induces
HMGA2	8091	Overexpression	Lung fibroblast	IMR-90	No	Oncogene-induced	Induces
HOPX	84525	Knockdown, Overexpression	Bronchial epithelial	HBEC, Y-BE	No	Oncogene-induced	Induces
HRAS	3265	Overexpression	Lung fibroblast	IMR-90, WI-38	No	Oncogene-induced	Induces
IFNG	3458	Overexpression	Umbilical vein endothelial	Primary cell	No	Unclear	Induces
IGFBP3	3486	Knockdown	Lung fibroblast	WI-38	No	Stress-induced	Induces
IGFBP5	3488	Knockout, Overexpression	Umbilical vein endothelial	Primary cell	No	Replicative	Induces
IGFBP7	3490	Knockdown	Foreskin fibroblast, Melanocyte	BJ, Primary cell	No	Oncogene-induced	Induces
IL1A	3552	Knockout	Umbilical vein endothelial	Primary cell	No	Unclear	Induces
IL1R1	3554	Knockdown	Foreskin fibroblast, Melanocyte	BJ, Primary cell	No	Oncogene-induced	Induces
IL6	3569	Knockdown	Fibroblast	TIG-3	No	Oncogene-induced, Stress-induced	Induces
ING1	3621	Overexpression	Foreskin fibroblast	HS68	No	Unclear	Induces
ING2	3622	Knockout, Overexpression	Lung fibroblast	MRC-5	No	Replicative	Induces
IRF1	3659	Knockdown	Foreskin fibroblast, Melanocyte	BJ, Primary cell	No	Oncogene-induced	Induces
IRF3	3661	Overexpression	Foreskin fibroblast	BJ	No	Unclear	Induces
ITGB4	3691	Knockdown	Umbilical vein endothelial	Primary cell	No	Unclear	Induces
ITPK1	3705	Overexpression	Embryonic kidney, Lung fibroblast	HEK293T, IMR-90, MRC-5	No	Unclear	Induces
ITPKB	3707	Overexpression	Embryonic kidney, Lung fibroblast	HEK293T, IMR-90, MRC-5	No	Unclear	Induces
ITPR1	3708	Knockdown	Mammary epithelial	HEC	No	Oncogene-induced	Induces
ITPR2	3709	Knockdown	Mammary epithelial	HEC	No	Oncogene-induced	Induces
ITPR3	3710	Knockdown	Mammary epithelial	HEC	No	Oncogene-induced	Induces
ITSN2	50618	Knockdown, Overexpression	Foreskin fibroblast, Lung fibroblast	HS68, WI-38	No	Stress-induced	Induces
JAK2	3717	Knockdown	Melanocyte	Primary cell	No	Unclear	Induces
KAT5	10524	Knockdown, Overexpression	Foreskin fibroblast	BJ	No	Oncogene-induced	Induces
KCNA1	3736	Knockdown	Mammary epithelial	Primary cell	No	Oncogene-induced	Induces
KEAP1	9817	Knockdown	Aortic endothelial	HAEC	No	Stress-induced	Induces
KNDC1	85442	Overexpression	Umbilical vein endothelial	HUVEC	No	Unclear	Induces
LATS2	26524	Knockout	Ovarian epithelial	Primary cell	No	Replicative	Induces
LAYN	143903	Knockdown	Mammary fibroblast	HMF3A	No	Unclear	Induces
LIMK1	3984	Overexpression	Embryonic kidney, Lung fibroblast	HEK293T, IMR-90, MRC-5	No	Unclear	Induces
MAP2K1	5604	Overexpression	Intestinal epithelial	HIEC-6	No	Unclear	Induces
MAP2K2	5605	Overexpression	Lung fibroblast	IMR-90	No	Oncogene-induced	Induces
MAP2K7	5609	Overexpression	Embryonic kidney, Lung fibroblast	HEK293T, IMR-90, MRC-5	No	Unclear	Induces
MAP3K5	4217	Mutation, Overexpression	Umbilical vein endothelial	HUVEC	No	Stress-induced	Induces
MAP3K6	9064	Overexpression	Embryonic kidney, Lung fibroblast	HEK293T, IMR-90, MRC-5	No	Unclear	Induces
MAP3K7	6885	Overexpression	Embryonic kidney, Lung fibroblast	HEK293T, IMR-90, MRC-5	No	Unclear	Induces
MAPK1	5594	Knockdown	Lung fibroblast	IMR-90	No	Oncogene-induced	Induces
MAPK12	6300	Overexpression	Embryonic kidney, Lung fibroblast	HEK293T, IMR-90, MRC-5	No	Unclear	Induces
MAPK14	1432	Knockout	Lung fibroblast	MRC-5, WI-38	No	Replicative, Stress-induced	Induces
MAPKAPK5	8550	Knockout	Foreskin fibroblast	BJ	No	Oncogene-induced	Induces
MAST1	22983	Overexpression	Embryonic kidney, Lung fibroblast	HEK293T, IMR-90, MRC-5	No	Unclear	Induces
MATK	4145	Overexpression	Embryonic kidney, Lung fibroblast	HEK293T, IMR-90, MRC-5	No	Unclear	Induces
MAVS	57506	Knockdown	Bone marrow mesenchymal stem	Primary cell	No	Unclear	Induces
MCRS1	10445	Overexpression	Foreskin fibroblast, Lung fibroblast, Mammary epithelial	H184B5F5/M10, HS68, IMR-90	No	Unclear	Induces
MCU	90550	Knockdown	Endothelial	Primary cell	No	Oncogene-induced	Induces
MEN1	4221	Knockdown	Foreskin fibroblast, Melanocyte	BJ, Primary cell	No	Oncogene-induced	Induces
MEOX1	4222	Overexpression	Umbilical vein endothelial	HUVEC	No	Unclear	Induces
MME	4311	Knockdown, Overexpression	Mammary epithelial	MCF-10A	No	Oncogene-induced, Stress-induced	Induces
MOB3A	126308	Overexpression	Embryonic kidney, Lung fibroblast	HEK293T, IMR-90, MRC-5	No	Unclear	Induces
MORC3	23515	Overexpression	Lung fibroblast	WI-38	No	Unclear	Induces
MOS	4342	Overexpression	Lung fibroblast	MRC-5	No	Oncogene-induced	Induces
MVK	4598	Overexpression	Embryonic kidney, Lung fibroblast	HEK293T, IMR-90, MRC-5	No	Unclear	Induces
NADK	65220	Overexpression	Embryonic kidney, Lung fibroblast	HEK293T, IMR-90, MRC-5	No	Unclear	Induces
NCAPH2	29781	Mutation, Overexpression	Lung fibroblast	IMR-90	No	Oncogene-induced	Induces
NDST2	8509	Knockdown	Lung fibroblast	IMR-90	No	Oncogene-induced	Induces
NEK4	6787	Knockdown	Foreskin fibroblast	BJ	No	Replicative, Stress-induced	Induces
NF2	4771	Knockdown	Foreskin fibroblast, Melanocyte	BJ, Primary cell	No	Oncogene-induced	Induces
NOLC1	9221	Knockdown, Overexpression	Lung fibroblast	2BS	No	Unclear	Induces
NOTCH1	4851	Overexpression	Esophageal keratinocyte	EPC1, EPC1-hTERT, EPC2-hTERT	No	Oncogene-induced	Induces
NOX1	27035	Overexpression	Pulmonary artery endothelial	Primary cell	No	Unclear	Induces
NOX4	50507	Knockdown, Overexpression	Breast epithelial, Lung fibroblast	MCF-12A, TIG-3	No	Oncogene-induced	Induces
NQO1	1728	Knockdown	Foreskin fibroblast, Lung fibroblast	2BS, BJ, IMR-90	No	Oncogene-induced	Induces
NRAS	4893	Mutation, Overexpression	Foreskin melanocyte	Primary cell	No	Oncogene-induced	Induces
NUAK1	9891	Knockout, Overexpression	Lung fibroblast	WI-38	No	Unclear	Induces
OGT	8473	Knockdown, Overexpression	Lung fibroblast	IMR-90	No	Oncogene-induced	Induces
PAK2	5062	Knockdown, Overexpression	Lung fibroblast	IMR-90	No	Oncogene-induced	Induces

PAK4	10298	Overexpression	Embryonic kidney, Lung fibroblast	HEK293T, IMR-90, MRC-5	No	Unclear	Induces
PBRM1	55193	Knockdown	Foreskin fibroblast	BJ	No	Replicative	Induces
PCGF2	7703	Knockout, Overexpression	Foreskin fibroblast, Lung fibroblast	BJ, MRC-5, WI-38	No	Unclear	Induces
PDCD10	11235	Knockout	Coronary artery endothelial, Lung fibroblast	IMR-90, Primary cell	No	Oncogene-induced, Replicative	Induces
PDGFB	5155	Overexpression	Dermal fibroblast	Primary cell	No	Oncogene-induced	Induces
PDIK1L	149420	Overexpression	Embryonic kidney, Lung fibroblast	HEK293T, IMR-90, MRC-5	No	Unclear	Induces
PDPK1	5170	Overexpression	Embryonic kidney, Lung fibroblast	HEK293T, IMR-90, MRC-5	No	Unclear	Induces
PI4KB	5298	Knockdown	Lung fibroblast	IMR-90	No	Oncogene-induced	Induces
PIK3R5	23533	Overexpression	Embryonic kidney, Lung fibroblast	HEK293T, IMR-90, MRC-5	No	Unclear	Induces
PIM1	5292	Knockdown, Overexpression	Foreskin fibroblast, Lung fibroblast	2BS, BJ, WI-38	No	Oncogene-induced, Replicative	Induces
PIN1	5300	Knockdown, Overexpression	Vascular smooth muscle	Primary cell	No	Replicative	Induces
PLA2G2A	5320	Overexpression	Lung fibroblast	WI-38	No	Replicative	Induces
PLA2R1	22925	Knockout, Overexpression	Lung fibroblast	IMR-90, WI-38	No	Replicative, Stress-induced	Induces
PML	5371	Overexpression	Lung fibroblast	WI-38	No	Oncogene-induced	Induces
PMVK	10654	Overexpression	Embryonic kidney, Lung fibroblast	HEK293T, IMR-90, MRC-5	No	Unclear	Induces
PPARG	5468	Knockdown	Lung fibroblast	2BS, WI-38	No	Replicative	Induces
PRKCD	5580	Knockdown, Overexpression	Lung fibroblast	TIG-1	No	Replicative	Induces
PRKD1	5587	Knockdown, Overexpression	Lung fibroblast	IMR-90	No	Oncogene-induced	Induces
PTGS2	5743	Overexpression	Foreskin fibroblast	NHDF	No	Replicative, Stress-induced	Induces
PTTG1	9232	Overexpression	Foreskin fibroblast, Lung fibroblast	BJ, IMR-90, WI-38	No	Unclear	Induces
RAF1	5894	Overexpression	Lung fibroblast	IMR-90	No	Oncogene-induced	Induces
RAP1GAP	5909	Knockdown	Foreskin fibroblast, Melanocyte	BJ, Primary cell	No	Oncogene-induced	Induces
RARB	5915	Overexpression	Dermal fibroblast	Primary cell	No	Oncogene-induced	Induces
RB1	5925	Knockdown	Osteoblast	Primary cell	No	Stress-induced	Induces
RBPJ	3516	Knockdown	Esophageal keratinocyte	EPC1-hTERT, EPC2-hTERT	No	Oncogene-induced	Induces
RNASEL	6041	Knockout, Overexpression	Lung fibroblast	WI-38	No	Replicative	Induces
ROMO1	140823	Knockdown, Overexpression	Lung fibroblast	IMR-90	No	Replicative	Induces
RPS6KB1	6198	Mutation, Overexpression	Umbilical vein endothelial	HUVEC	No	Unclear	Induces
RPTOR	57521	Knockdown	Nucleus pulposus	Primary cell	No	Unclear	Induces
SERPINB2	5055	Overexpression	Lung fibroblast	IMR-90	No	Unclear	Induces
SERPINE1	5054	Knockdown	Foreskin fibroblast	BJ, Primary cell	No	Replicative	Induces
SFRP1	6422	Knockdown	Lung fibroblast	IMR-90	No	Stress-induced	Induces
SIAH1	6477	Knockdown	Lung fibroblast	MRC-5	No	Replicative	Induces
SIK1	150094	Overexpression	Embryonic kidney, Lung fibroblast	HEK293T, IMR-90, MRC-5	No	Unclear	Induces
SIN3B	23309	Knockout, Overexpression	Lung fibroblast	IMR-90	No	Unclear	Induces
SLC13A3	64849	Overexpression	Lung fibroblast, Renal tubular	MRC-5, WI-38	No	Stress-induced	Induces
SLC31A2	1318	Knockdown	Lung fibroblast	IMR-90	No	Oncogene-induced	Induces
SLC5A2	6524	Knockdown	Proximal tubular cell	Primary cell	No	Stress-induced	Induces
SMAD3	4088	Knockdown	Mammary epithelial	Primary cell	No	Stress-induced	Induces
SMURF2	64750	Overexpression	Foreskin fibroblast, Lung fibroblast, Skin fibroblast	BJ, WI-38, WS1	No	Replicative	Induces
SOCS1	8651	Knockdown, Overexpression	Lung fibroblast	IMR-90	No	Oncogene-induced, Replicative	Induces
SOD2	6648	Overexpression	Prostate epithelial	M12	No	Unclear	Induces
SORBS2	8470	Overexpression	Fibroblast, Keratinocyte	Primary cell	No	Unclear	Induces
SP1	6667	Overexpression	Lung fibroblast	2BS	No	Unclear	Induces
SPI1	6688	Overexpression	Foreskin fibroblast, Hematopoietic stem progenitor, Lung fibroblast	BJ, Primary cell, WI-38	No	Oncogene-induced	Induces
SPOP	8405	Knockdown, Mutation, Overexpression	Lung fibroblast	IMR-90	No	Oncogene-induced	Induces
STAG2	10735	Knockout	Foreskin fibroblast	BJ	No	Replicative	Induces
STAT1	6772	Knockdown	Glomerular mesangial cell	Primary cell	No	Unclear	Induces
STAT3	6774	Overexpression	Fibroblast	TIG-3	No	Oncogene-induced	Induces
STAT5A	6776	Knockout, Overexpression	Fibroblast	Primary cell	No	Oncogene-induced	Induces
STAT5B	6777	Mutation	Lung fibroblast	IMR-90	No	Unclear	Induces
STK32C	282974	Overexpression	Embryonic kidney, Lung fibroblast	HEK293T, IMR-90, MRC-5	No	Unclear	Induces
STK4	6789	Overexpression	Fetal lung fibroblast, Umbilical cord fibroblast	HUC-F2, TIG-1	No	Unclear	Induces
STK40	83931	Overexpression	Embryonic kidney, Lung fibroblast	HEK293T, IMR-90, MRC-5	No	Unclear	Induces
SUMO3	6612	Overexpression	Embryonic kidney	HEK294	No	Unclear	Induces
TGFB1I1	7041	Knockout, Overexpression	Fibroblast	KMST-6, SUSM-1	No	Unclear	Induces
TLR10	81793	Knockdown	Embryonic kidney, Lung fibroblast	HEK293T, IMR-90	No	Oncogene-induced	Induces
TLR2	7097	Knockdown, Overexpression	Embryonic kidney, Lung fibroblast	HEK293T, IMR-90	No	Oncogene-induced	Induces
TLR3	7098	Overexpression	Fibroblast, Keratinocyte	Primary cell	No	Unclear	Induces
TNFSF15	9966	Knockout, Overexpression	Endothelial progenitor	Primary cell	No	Replicative	Induces
TOM1	10043	Overexpression	Lung fibroblast	2BS	No	Unclear	Induces
TOP1	7150	Knockout	Lung fibroblast	IMR-90, WI-38	No	Oncogene-induced	Induces
TP53BP1	7158	Knockout	Retinal pigment epithelial	RPE-1	No	Unclear	Induces
TP53BP2	7159	Knockdown	Lung fibroblast	IMR-90	No	Oncogene-induced	Induces
TP53INP1	94241	Knockdown	Human embryonic lung diploid fibroblast	WI-38	No	Stress-induced	Induces
TP63	8626	Knockout	Foreskin fibroblast	BJ	No	Oncogene-induced	Induces
TRIM28	10155	Knockdown	Embryonic kidney, Lung fibroblast	HEK293T, IMR-90	No	Oncogene-induced	Induces
TXNIP	10628	Knockout, Overexpression	Lung fibroblast	2BS	No	Oncogene-induced, Replicative	Induces
TYK2	7297	Overexpression	Embryonic kidney, Lung fibroblast	HEK293T, IMR-90, MRC-5	No	Unclear	Induces
ULK3	25989	Overexpression	Lung fibroblast	IMR-90	No	Unclear	Induces
USP28	57646	Knockout	Retinal pigment epithelial	RPE-1	No	Unclear	Induces
VCAN	1462	Knockdown	Umbilical vein endothelial, Vascular smooth muscle	HUVEC, Primary cell	No	Stress-induced	Induces
WNT16	51384	Knockdown	Lung fibroblast	MRC-5	No	Replicative	Induces

XAF1	54739	Knockdown, Overexpression	Dermal microvascular endothelial	Primary cell	No	Stress-induced	Induces
XPO1	7514	Overexpression	Fibroblast	Primary cell	No	Unclear	Induces
