# Supplementary material for: Senescence‐Driven Remodeling Defines an Aggressive and Immunomodulatory Subtype of Endometriosis
Source: Aging Cell. 2026 Mar 27;25(4):e70463. doi: 10.1111/acel.70463 (PMC13140525; doi:10.1111/acel.70463)
Supplement: Supplementary file 4 — Table S3: Molecular docking parameters. [file ACEL-25-e70463-s004.pdf]

Supplementary Table 3. Molecular docking parameters.

Protein	Ligand	Vina score	Cavity volume (Å3)
PAK4	Stigmasterol	-8.7	4557
HMGA1	Stigmasterol	-5.9	36
NCAPH2	Stigmasterol	-7.4	914
SOCS1	Stigmasterol	-7.7	350
